# Occupancy by key transcription factors is a more accurate predictor of enhancer activity than histone modifications or chromatin accessibility

**DOI:** 10.1186/s13072-015-0009-5

**Published:** 2015-04-23

**Authors:** Nergiz Dogan, Weisheng Wu, Christapher S Morrissey, Kuan-Bei Chen, Aaron Stonestrom, Maria Long, Cheryl A Keller, Yong Cheng, Deepti Jain, Axel Visel, Len A Pennacchio, Mitchell J Weiss, Gerd A Blobel, Ross C Hardison

**Affiliations:** Center for Comparative Genomics and Bioinformatics, Department of Biochemistry and Molecular Biology, The Pennsylvania State University, 304 Wartik Laboratory, University Park, PA 16802 USA; Bioinformatics Core, Department of Computational Medicine and Bioinformatics, University of Michigan, Ann Arbor, MI 48109-2218 USA; Division of Hematology, The Children’s Hospital of Philadelphia, 3401 Civic Center Boulevard, Philadelphia, PA 19104 USA; Perelman School of Medicine at the University of Pennsylvania, 415 Curie Boulevard, Philadelphia, PA 19104 USA; Department of Genetics, Mail Stop-5120, Stanford University, Stanford, CA 94305 USA; Genomics Division, Lawrence Berkeley National Laboratory, 1 Cyclotron Road, Mailstop 84-171, Berkeley, CA 94720 USA; DOE Joint Genome Institute, 2800 Mitchell Drive, Walnut Creek, CA 94598 USA; Department of Hematology, St Jude Children’s Research Hospital, 262 Danny Thomas Place, Memphis, TN 38105 USA

**Keywords:** Functional genomics, Gene regulation, TAL1, GATA1, Histone modifications, Enhancer assay

## Abstract

**Background:**

Regulated gene expression controls organismal development, and variation in regulatory patterns has been implicated in complex traits. Thus accurate prediction of enhancers is important for further understanding of these processes. Genome-wide measurement of epigenetic features, such as histone modifications and occupancy by transcription factors, is improving enhancer predictions, but the contribution of these features to prediction accuracy is not known. Given the importance of the hematopoietic transcription factor TAL1 for erythroid gene activation, we predicted candidate enhancers based on genomic occupancy by TAL1 and measured their activity. Contributions of multiple features to enhancer prediction were evaluated based on the results of these and other studies.

**Results:**

TAL1-bound DNA segments were active enhancers at a high rate both in transient transfections of cultured cells (39 of 79, or 56%) and transgenic mice (43 of 66, or 65%). The level of binding signal for TAL1 or GATA1 did not help distinguish TAL1-bound DNA segments as active *versus* inactive enhancers, nor did the density of regulation-related histone modifications. A meta-analysis of results from this and other studies (273 tested predicted enhancers) showed that the presence of TAL1, GATA1, EP300, SMAD1, H3K4 methylation, H3K27ac, and CAGE tags at DNase hypersensitive sites gave the most accurate predictors of enhancer activity, with a success rate over 80% and a median threefold increase in activity. Chromatin accessibility assays and the histone modifications H3K4me1 and H3K27ac were sensitive for finding enhancers, but they have high false positive rates unless transcription factor occupancy is also included.

**Conclusions:**

Occupancy by key transcription factors such as TAL1, GATA1, SMAD1, and EP300, along with evidence of transcription, improves the accuracy of enhancer predictions based on epigenetic features.

**Electronic supplementary material:**

The online version of this article (doi:10.1186/s13072-015-0009-5) contains supplementary material, which is available to authorized users.

## Background

Accurate identification of *cis*-regulatory modules (CRMs), such as enhancers, is essential for understanding mechanisms of gene regulation, modeling regulation during differentiation, and interpreting the effects of genetic variants associated with complex traits. Challenges to meeting this goal are formidable [1]. Most enhancers are in the portion of the genome that does not code for proteins, and thus the search space is enormous. No clear grammar for identifying enhancers with a high success rate has been discovered, and examination of the primary DNA sequence has limited power for identifying enhancers [2]. Evidence of strong evolutionary constraint [3-5] has consistently revealed enhancers, but these are enriched for certain classes of genes and tissues [6-8] and do not capture the full spectrum of regulatory regions [9,10]. Application of machine learning to find discriminatory patterns in alignments of known regulatory regions has also been successful [11-13]. Other methods for predicting enhancers based on conservation of noncoding regions have been less successful [14], illustrating a need for improving enhancer prediction by methods inxc addition to conservation or alignment-based approaches.

Understanding both biochemical mechanisms of enhancement and evolution of enhancers provides a basis for more integrated approaches for their prediction. Enhancers are clusters of transcription factor (TF) binding site motifs in the DNA, and they are active when the TFs are bound [15]. The complex of bound proteins tends to interact with co-activators such as EP300 [16], and the TF-bound enhancer is flanked by nucleosomes with characteristic histone modifications, such as H3K4 monomethylation (H3K4me1) and H3K27 acetylation (H3K27ac) [17-19]. These epigenetic features (proteins or modifications, including those in chromatin, that lie on top of the DNA sequence) have been used for predictions of enhancers. For instance, the presence of H3K4me1 with little or no trimethylation (H3K4me3) predicted enhancers with good accuracy [20,21], enrichment by H3K27ac separated active enhancers from poised and inactive enhancers [18,22], and the presence of EP300 identified heart enhancers with high accuracy (75%) [8]. The combination of H3K4me1 plus EP300 was highly accurate (70% to 86%) for identifying enhancers in melanocytes [23]. Using tissue-specific TFs such as GATA1 in erythroid cells [24] and MYOD in muscle cells [25] to predict enhancers had a good success rate (40% to 50%) but lower than that for co-activators. Formal approaches to integrating multiple features for enhancer prediction include a genome segmentation based on multiple histone modifications and other features, utilizing a hidden Markov model framework [26]. Two recent studies using massively parallel reporter assays (MPRAs) found that 23% to 42% [27] or 26% [28] of these predicted enhancers were significantly active. Another approach used a discriminatory machine-learning approach to integrate multiple datasets and data types to predict and validate developmental enhancers [29].

While the power of epigenetic approaches for enhancer prediction is clear, the features with the strongest contributions to predictive accuracy are not known. Despite the importance of TF occupancy in the mechanism of enhancement, it has not been the focus of recent methods for enhancer prediction, perhaps because of the tissue-specificity of many TFs that play a role in enhancement. Nevertheless, many high-quality datasets of TF occupancy are being determined genome-wide, many TFs are active in multiple tissues (albeit not ubiquitously), and co-activators such as EP300 are active in a large number of tissues. Thus, it is important to examine the role of TFs; this is most informative within a tractable developmental system and with multiple assays for enhancement.

To address this need, we focused initially on occupancy by TAL1, since this TF plays multiple key roles in hematopoiesis and is needed for differentiation of erythroid progenitor cells into maturing erythroblasts [30]. Experiments using conditional *Tal1* knockout mutants and rescue show that TAL1 is required for both specification and differentiation of erythroid and megakaryocytic cells [31]. Furthermore, the co-binding of TAL1 with GATA1 is strongly associated with gene induction [32-35]. Thus, we began with high-quality datasets on TAL1 occupancy in a mouse cell line model for maturing erythroblasts [36,37] as a single-factor predictor of erythroid CRMs. Remarkably, a majority of the DNA segments predicted as CRMs by this one factor and tested by reporter gene assays in either transfected cells in culture or transgenic mouse embryos were active as enhancers. Using ChIP-seq data for multiple epigenetic features [10,38], we evaluated the contributions to enhancement of these features on a meta-analysis of 273 DNA segments tested for enhancement. Multiple features contributed to accurate prediction of enhancer activity at TAL1-bound DNA segments, and the presence together of TAL1, GATA1, EP300, SMAD1, H3K4 methylation, H3K27ac, and CAGE tags at DNase hypersensitive sites correctly predicted erythroid enhancers in over 80% of cases. In contrast, DNA segments in chromatin that is accessible or marked with enhancer-associated histone modifications but lacking binding by key TFs are rarely active as enhancers.

## Results

### Occupancy by TAL1 is a strong predictor of enhancer activity

**Figure 1:**
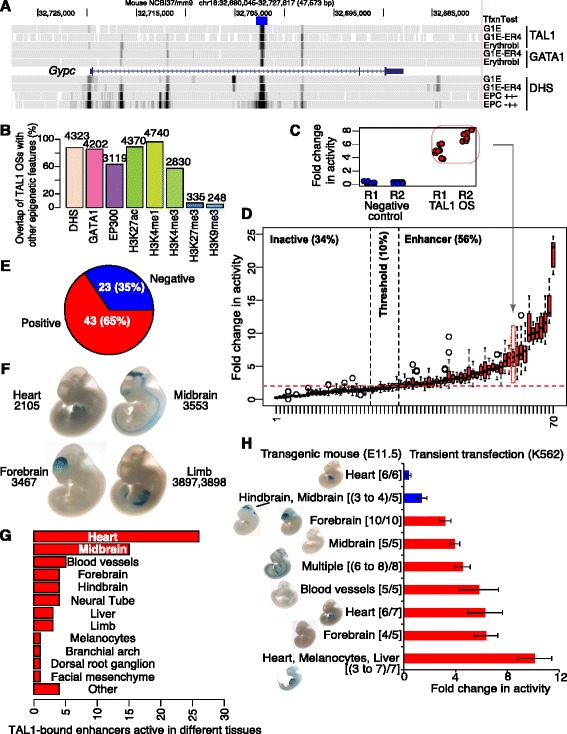
Genome-wide prediction of TAL1 OSs as enhancers. **(A)** Epigenetic marks overlapping a TAL1 peak within *Gypc*. Tracks displayed on the UCSC Genome Browser [109] show, in descending order, the DNA segment tested for enhancer activity, occupancy by TAL1 and GATA1, the gene model, DNase hypersensitive sites in G1E cells, G1E-ER4 cells treated with estradiol, and mouse primary fetal liver-derived early erythroid progenitors (EPC CD117+, CD71+, TER119-) and differentiating erythroblasts (EPC CD117-, CD71+, TER119+). **(B)** Overview of ChIP-seq data for epigenetic features at TAL1 peaks. **(C**, **D)** Erythroid enhancer activity of TAL1 OSs in a transient transfection assay. **(C)** Biological replicates (two different days of transfection, Rep1 and Rep2) and technical replicates (eight for each biological replicate) of the enhancer assays of a negative control vector and an expression vector containing TAL1 OS from the *Gypc* intron. **(D)** Enhancer assay results for 70 TAL1 OSs, ordered by activity. The distribution of results for each TAL1 OS is shown as a box plot, with the internal line indicating the median value. Boxes for inactive TAL1 OSs are shaded blue, those in the threshold zone are pink, and those with activity are shaded red. **(E**, **F**, **G)** Tissue-specific enhancer activities of TAL1 OSs in transgenic mouse assays. **(E)** Partitions of 66 TAL1 OSs by enhancer activity. **(F)** Four examples of whole mouse embryos with *in vivo* enhancer activity at E11.5. **(G)** Distribution of tissues showing enhancement by the TAL1 OSs. For TAL1 OSs active in multiple tissues, each tissue was counted for the distribution. **(H)** Comparison of the results of the two enhancer assays on nine TAL1 OSs. Stained mouse images are from the VISTA Enhancer Browser.

We began with the set of 4,915 DNA segments determined by ChIP-seq to be bound by TAL1 (TAL1 occupied DNA segments or TAL1 OSs) (Table 1; Additional file 1: Table S1) in G1E-ER4 cells treated with beta-estradiol, which are a model for erythroblasts differentiating in response to restoration of GATA1 [39,40]. As expected [41-43], the TAL1 OSs overlapped with other epigenetic features suggestive of regulatory function, illustrated by the example of a candidate enhancer in an intron of the gene *Gypc*, which encodes the erythroid membrane protein glycophorin C (Figure 1A). The vast majority of TAL1 OSs were in regions of accessible chromatin (DNase I hypersensitive sites, DHSs; [36,44]) containing histone modifications (HMs) associated with gene regulatory regions (H3K27ac [45], H3K4me1 [36], and/or H3K4me3 [36]; Figure 1B). Most peaks of TAL1 binding coincided with binding by GATA1 [34,36] and the coactivator EP300 [10,46] (Figure 1B). Only a small minority had the H3K27me3 [36] or H3K9me3 [36] modifications associated with gene repression (Figure 1B). The presence of the promoter-associated histone mark H3K4me3 [20,47] on the subset of TAL1 OSs that were close to transcription start sites (TSS) (478 were within 1 kb of a TSS; Additional file 2: Figure S1) showed that some TAL1 OSs are close to promoters. However, it is difficult to distinguish activity directly in a promoter *versus* in an enhancer that is located adjacent to the promoter. Hence, all the TAL1 OSs were considered candidates for enhancers, even though some could also be in DNA segments active as promoters. From the set of 4,915 DNA segments bound by TAL1, 70 (Additional file 1: Tables S2 and S3) were tested for enhancer activity after transient transfection in K562 cells, which have properties of erythroid and megakaryocytic cells [48]. The 70 were chosen randomly from eight clusters of TAL1 OSs characterized by additional epigenetic features, as described in the next section. Each TAL1 OS was tested for the ability to increase luciferase expression from a reporter gene in multiple experiments [12,49], with good reproducibility between both biological and technical replicates (Figure 1C; Additional file 2: Figure S2A). The results, presented as box plots summarizing all the data for each of the 70 tested TAL1 OSs (Figure 1D; values are in Additional file 1: Table S3), showed that 39 (56%) produced at least a twofold increase. Many were strongly active, with 26 giving at least a threefold increase, and the most active one generating a median effect of 23-fold. Activity of another 7 (10%) TAL1 OSs fell in a ‘threshold zone,’ which was less than the twofold needed to be declared an enhancer by this assay, but greater than 1.5-fold, which is over three standard deviations above the median of the negative controls. The remaining 24 TAL1 OSs were not active in this enhancer assay. While the transient transfection assay reveals the ability to increase expression from a plasmid that acquires some aspects of chromatin structure [50], the DNA does not integrate into chromosomes, and the activity is assayed in a single-cell type.

**Table 1:** **ChIP-seq datasets**

**Feature**	**Cell line**	**Number of peaks**	**Filename at UCSC Genome Browser or GEO series number**
TAL1	G1E-ER4 + E2	4,915	wgEncodePsuTfbsG1eer4e2Tal1ME0S129InputPk.broadPeak.gz
GATA1	G1E-ER4 + E2	13,123	wgEncodePsuTfbsG1eer4e2Gata1aME0S129InputPk.broadPeak.gz
EP300	MEL	31,342	wgEncodeSydhTfbsMelP300IggrabPkV2.narrowPeak.gz, wgEncodeSydhTfbsMelP300sc584IggrabPk.narrowPeak.gz
SMAD1	G1E-ER4 + E2 + BMP4	1,586	GSE29193
H3K4me1	G1E-ER4 + E2	105,231	wgEncodePsuHistoneG1eer4e2H3k04me1ME0S129InputPk.broadPeak.gz
H3K4me3	G1E-ER4 + E2	72,495	wgEncodePsuHistoneG1eer4e2H3k04me3ME0S129InputPk.broadPeak.gz
H3K27me3	G1E-ER4 + E2	53,587	wgEncodePsuHistoneG1eer4e2H3k27me3ME0S129InputPk.broadPeak.gz
H3K9me3	G1E-ER4 + E2	69,929	wgEncodePsuHistoneG1eer4e2H3k09me3ME0S129InputPk.broadPeak.gz
H3K27ac	G1E-ER4 + E2	31,535	GSE61349
DNaseI hypersensitivity	G1E-ER4 + E2	93,705	wgEncodePsuDnaseG1eer4S129ME0Diffd24hPkRep1.narrowPeak.gz

*Note*: G1E-ER4 + E2: cells treated with estradiol for 24 h.

We then turned to assays for tissue-specific enhancement in transgenic mouse embryos [5]. A large number of human and mouse candidate *cis*-regulatory modules, predicted by interspecies conservation of noncoding sequences [5], EP300 occupancy [7], or other features [51], have been tested for the ability to increase expression of a beta-galactosidase reporter gene driven by a minimal promoter in a tissue-specific manner at embryonic day 11.5 of mice. Of the 4,915 erythroid TAL1 OSs, 66 (mouse DNA fragments or their human orthologs) have been tested in the mouse transient transgenic assay, as recorded in the VISTA Enhancer Browser [52]. Remarkably, 43 of these (65%) were reproducibly active in one or more tissues, with the greatest number in heart and midbrain (Figure 1E-G; Additional file 1: Table S4).

Nine TAL1 OSs were tested in both assays. All nine were active in a distinct set of tissues, and seven of the nine were also active in the transient transfection assay (Figure 1H; Additional file 2: Figure S2B). Thus, our assessment of enhancer activity for the TAL1 OSs is robust, with a large majority of the tested DNA fragments active in both assays.

### Signal strength does not help distinguish TAL1 OSs active as enhancers

**Figure 2:**
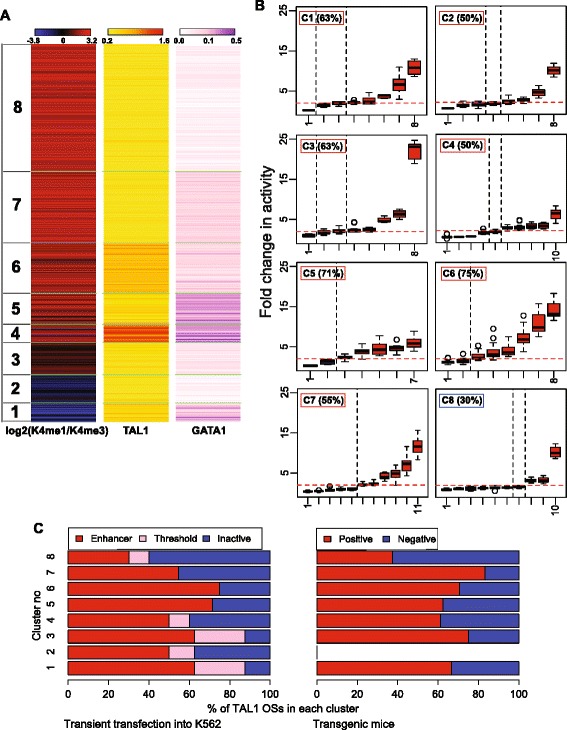
Clustering TAL1 OSs based on epigenetic features. **(A)** TAL1 OSs clustered by the ChIP-seq signals of H3K4me1 and H3K4me3 (log_2_ of the ratio), TAL1, and GATA1 (k-means clusters, *k* = 8). **(B)** Enhancer activities of TAL1 OSs tested by transient transfection assays in K562 cells, grouped in clusters by epigenetic features. The names of individual TAL1 OSs are given along the x-axis, and the percent active in each cluster is listed. The distinctive properties of each TAL1 OS cluster are summarized in the three colored bars, derived from Figure 2A. **(C)** Activities of TAL1 OSs grouped in clusters by epigenetic features, shown for both enhancer assays: transient transfection into K562 cells (*left*) and transgenic mice at E11.5 (*right*).

The TAL1 OSs share many features associated with enhancer activity (Figure 1B), and thus we searched for additional factors that could distinguish the enhancer-active ones from those that are inactive in these assays. We hypothesized that the level of signal for binding by known TFs or HMs associated with regulation could distinguish enhancer-active TAL1 OSs from inactive ones. Given the strong association of gene induction with co-occupancy by GATA1 with TAL1 [32-35], we computed the level of ChIP-seq signal for both GATA1 and TAL1 in each of the 4,915 TAL1 OSs. Note that all DNA segments have TAL1 signal above the threshold for peak calling, but some have GATA1 signals below such a threshold. For each TAL1 OS, the signal levels for H3K4me1 and H3K4me3 HMs were also computed and expressed as the log base 2 of the ratio of monomethylation to trimethylation of H3K4 [20,47]. The resulting data matrix was then organized into clusters consisting of fairly homogenous combinations of the three features using k-means clustering, *k* = 8 (Figure 2A; Additional file 1: Table S5). The clusters had distinctive features that may be expected to correlate with differences in enhancer activity. For example, cluster 1 had a higher level of H3K4 trimethylation than monomethylation, characteristic of regions proximal to promoters, and indeed, a majority of TAL1 OSs in cluster 1 were within 1 kb of a TSS (Additional file 2: Figure S3A). Clusters 3 to 8 had properties associated with enhancers, including higher levels of H3K4 monomethylation than trimethylation (Figure 2A) and genomic location distal to the closest TSS (Additional file 2: Figure S3A). DNA segments in clusters 1, 4, and 5 had high signals for GATA1 occupancy and thus would be considered particularly strong candidates for erythroid enhancers. Almost all TAL1 OSs in each cluster were in DHSs, and a majority were also bound by EP300, with the exception of cluster 2 (Additional file 2: Figure S3C). Likewise, the currently known erythroid CRMs [36] were distributed among all clusters, except for clusters 2 and 8 (Additional file 2: Figure S3B).

Our hypothesis predicts that the rate of finding active enhancers should vary significantly among the clusters. The 70 TAL1 OSs tested for enhancer activity in transient transfections were randomly chosen from each of the eight clusters, with 7 to 11 DNA segments from each (Additional file 1: Tables S3 and S5). Unexpectedly, the frequency of positives did not differ dramatically among most of the clusters (Figure 2B), ranging from 50% to 75% for almost all the clusters. The only exception is cluster 8 with only a 30% success rate. Notably, the TAL1 OSs in this cluster have a very low level of GATA1 binding signal, indicating that TAL1 OSs that lack GATA1 are less likely to be enhancers. Surprisingly, high levels of TAL1 and GATA1 signal did not correlate with higher frequency of enhancer activity. Cluster 4 had the highest levels of TAL1 and GATA1, yet its rate of positives (50%) was lower than that of several other clusters. Notably, a substantial majority of TAL1 OSs with lower ratios of H3K4me1/H3K4me3 (clusters 1 and 2) and proximity to a TSS (cluster 1) were active as enhancers in the transient transfection assay. While many TAL1 OSs in cluster 1 were expected to be promoters, the results show that they also contain promoter-proximal enhancers. Cluster 2 also had a preponderance of H3K4me3 over H3K4me1, but only 28% of the TAL1 OSs in this cluster were within 1 kb of an annotated TSS. Half of the tested TAL1 OSs in cluster 2 were active as enhancers.

Examination of the level of TF binding signal or HM without regard to the clustering patterns confirmed that, *for these DNA segments bound by TAL1*, signal strength does not correlate with activity. The correlation coefficient R ranges from negative values for TAL1 and H3K4me1 signal to a low positive value for GATA1 signal (Additional file 2: Figure S4A). The three activity categories (active, threshold, and inactive) showed similar distributions of signal intensity for TAL1 binding, GATA1 binding, and the ratio of H3K4me1 to H3K4me3 (Additional file 2: Figure S4B). This absence of a correlation between enhancer activity binding signal strength persists even when the signals are evaluated at DNA intervals called as peaks of TF binding (Additional file 2: Figure S4A for TAL1 and S4C for GATA1). Likewise, the G + C content of the tested TAL1 OSs DNA segments had little discriminatory power; each activity class had similar GC content (Additional file 2: Figure S3D). The presence of EP300 at a TAL1 OS did help distinguish active *versus* inactive in this assay but not dramatically. The fraction of enhancer-active TAL1 OSs with EP300 (80%) was larger than the fraction of inactive TAL1 OSs with EP300 (61%) (Additional file 2: Figure S3E). Moreover, among the TAL1 OSs in cluster 8, none of the inactive regions were bound by EP300.

The limited effect of signal levels on success of predicting enhancers (conditional on TAL1 occupancy) was also seen for the transient transgenic mouse assays. The TAL1 OSs determined to be active enhancers in each of the two assays were distributed similarly among the eight clusters (Figure 2C; Additional file 1: Table S4). This includes the lower frequency of successful predictions when TAL1 is bound with little or no GATA1 (cluster 8).

### Greater contribution of TF binding than HMs to successful prediction of enhancers

In order to evaluate the contribution of multiple epigenetic features to the likelihood of a DNA segment being an enhancer, we expanded our study to include 273 DNA segments evaluated for enhancer activity by transfection into K562 cells in this laboratory [12,24], including the 70 TAL1 OSs just described. This larger meta-analysis is not restricted to any particular TF occupancy or conservation pattern, and it includes several DNA segments previously used as negative controls. Each of the 273 DNA segments were annotated by the presence or absence of ten features, specifically DHSs, binding by TAL1 or GATA1, or presence of H3K4me1, H3K4me3, H3K27ac, H3K27me3, or H3K9me3 (all in G1E-ER4 cells induced for 24 h with estradiol), binding by EP300 in MEL cells [46], or presence of CAGE (cap analysis of gene expression) tags [53]. The level of enhancer activity (fold change) and category (active, threshold, or inactive) was also recorded in the data matrix (Additional file 1: Table S6).

**Figure 3:**
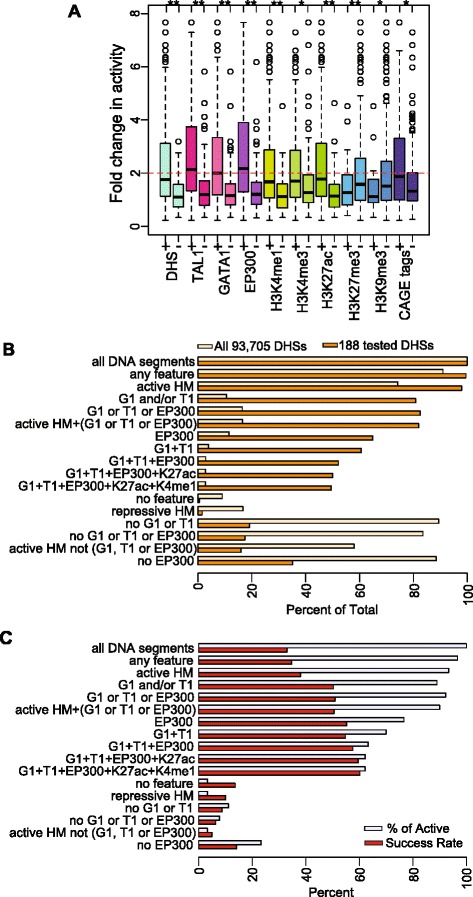
Contributions of TF binding *versus* histone modification enrichment to enhancer activity. **(A)** Distributions of enhancer activities of 273 DNA segments marked by each feature individually. The asterisks indicate statistically significant difference in activity between the presence and absence of the features. **(B)** Proportions of DHSs in 24-h-induced G1E-ER4 cells (total of 93,705) and tested DNA fragments that overlap DHSs (total of 188) with each feature combination. **(C)** The percentage of active enhancers captured by each set of features and the success rate of tested DNA segments (total of 273) with each feature combination.

We confirmed that four commonly used epigenetic predictors of enhancers, DHSs, H3K4me1, H3K27ac, and occupancy by the co-activator EP300, were strongly positively associated with enhancement (Figure 3A). The range of activities is consistently and significantly higher for DNA segments with these features than those without them, and EP300 had the strongest effect. Importantly, the presence of either TAL1 or GATA1 was also highly predictive of enhancer activity, with median activity levels comparable to that for EP300-positive DNA segments (Figure 3A; Additional file 1: Tables S6 and S7). The presence of H3K4me3 was associated with higher activity in this assay, perhaps reflecting promoter-proximal enhancers. CAGE tags also were associated with enhancer activity (Figure 3A), and the frequency of observing CAGE tags at active enhancers (49%) was almost twice that for non-active DNA segments (27%; Additional file 2: Figure S5). Moreover, the median values of enhancer activities for TF-bound DNA segments (twofold change in activity or greater; Figure 3A) were consistently higher than those for DNA segments with active HMs, DHSs, or CAGE tags (ranging from 1.67 to 1.87). As expected, the repressive HMs were not associated with enhancer activity (Figure 3A; Additional file 1: Table S7).

In order to examine the prevalence of these epigenetic features across the accessible chromatin landscape, we determined their presence or absence in the 93,705 DHSs ascertained in 24-h-induced G1E-ER4 cells [36,54]. Only a small fraction of DHS were bound by the TFs associated with enhancement. Specifically, 11% (10,012) were bound by GATA1 and/or TAL1, and 16% (15,456) were bound by GATA1, TAL1, and/or EP300 (Figure 3B). In contrast, a much higher proportion (74%) of the DHSs carried at least one active histone mark (H3K27ac, H3K3me1, or H3K3me3). A majority (58%) of DHSs were in chromatin with an activity-associated HM but not bound by any of the three TFs. Reflecting the use of prior knowledge about enhancer-associated features in selecting DNA segments for experimental tests, a larger proportion of DHSs actually tested for enhancement were bound by TFs (Figure 3B). Despite the small fraction of all DHS bound by the examined TFs, such binding was effective at capturing most (90% to 95%) of the 90 active enhancers in the set of 273 tested DNA segments (Figure 3C). Furthermore, the rate at which TF-bound DNA segments were active as enhancers was over 50%. In contrast, activity-associated HMs in the absence of TF binding did not capture known enhancers, nor were they good predictors of activity.

**Figure 4:**
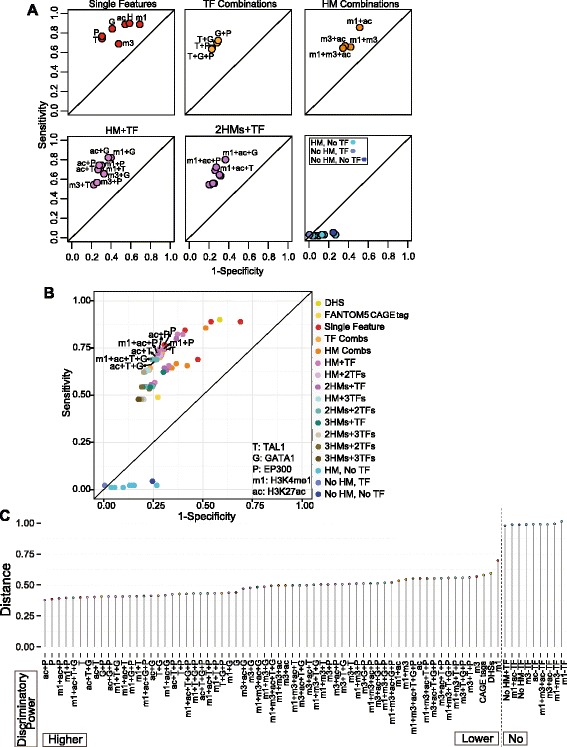
Meta-analysis of contributions of epigenetic features to enhancer activity. The sensitivity and specificity of epigenetic features singly and in combination for prediction of enhancer activity were evaluated for 273 DNA segments tested in transient transfection assays. The results are displayed as a receiver-operator characteristic (ROC) graph. The graphs in **(A)** show the results for informative groups of features, and **(B)** shows the results for all combinations of features. Abbreviations and color code are defined in panel **(B)**. The top eight discriminators with the best performance (dots in the upper left) are labeled in **(B)**. **(C)** Dot plot illustrating the distance of 74 feature and feature combinations to the point with the best discriminator performance (Sn = 1 and Sp = 1).

We then computed the sensitivity (Sn) and specificity (Sp) of features, individually and in combination, for predicting enhancers active in the transient transfection assay. For 74 combinations of epigenetic features (including individual features; Additional file 1: Table S8), the tested DNA segments that had a given set of features were considered the positive predictions for enhancer activity, and the remaining tested DNA fragments were considered negatives. These predictions were then evaluated by the results of the enhancer assay, thereby separating the positive predictions into true and false positives and the negative predictions into true and false negatives. Thus, we could calculate the Sn and Sp for enhancer prediction by each set of features, utilizing the information from all 273 tested DNA fragments for each set of features. Unless explicitly stated (for example, HMs without TF binding), a DNA segment was evaluated as having a given feature regardless of the status of other features. We displayed the results in ROC (receiver operating characteristic) graphs (Figure 4), so that the best discriminators generated points in the upper left of the graph, whereas feature(s) with low discriminatory power generated points along or to the right of the diagonal. The sets of features were described by the minimum requirement for inclusion, so that, for example, DNA segments with both TAL1 and H3K27ac were a subset of the group of DNA segments with TAL1.

We found that TF binding was a better predictor of enhancer activity than activity-associated HMs, primarily because of the higher specificity of the former (Figure 4A); this was seen both for individual features and combinations. The two best single discriminators were EP300 binding and TAL1 occupancy; they were almost indistinguishable in Sn (77% and 74%, respectively) and identical in Sp (69%). Combinations of TF binding and activity-associated HMs also had good performance. Considering histone modifications without regard to TF binding gave high Sn (0.89 for H3K4me1 or H3K27ac) but low Sp (0.31 for H3K4me1 and 0.46 for H3K27ac). Importantly, the presence of activity-associated HMs in the *absence* of TF binding performed very poorly, with a Sn of 0.01 to 0.02. The presence of TF binding in the absence of activity-associated HMs also gave a low Sn (0.02), but this arrangement was rare (only three DNA segments in the 273 examined). The ROC of all the examined feature combinations together shows that many combinations of TFs and HMs have good performance, forming a cluster in the upper left of the graph (Figure 4B). The discriminatory power of each feature and combination is better resolved in the dot plot (Figure 4C), which displays the distance of each (1-Sp, Sn) point from the ideal of (0,1). Over 15 features individually or in combination had an equivalently short distance (that is, good discriminatory power). Two of the strongest discriminators were binding by TAL1 or by EP300. Combining binding by either TF with HMs such as H3K27ac or H3K4me1 gave only a very small increase in power. Having H3K4me3 as a feature lowered the discriminatory power (Figure 4C). DHS or CAGE tags individually had some discriminatory power but considerably less than TF binding. Repeating the ROC analysis on the subset of 188 tested DNA segments that are also DHS gave similar results for the discriminatory power of these epigenetic features (Additional file 2: Figure S6; Additional file 1: Table S9). For the large majority of feature combinations, adding DHS had no impact on Sn or Sp, although for a small minority the Sp was increased.

**Figure 5:**
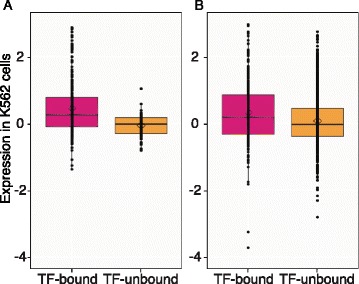
Association of TF binding with strength of enhancement in high-throughput enhancer assays. Distribution of expression levels of **(A)** 320 DNA segments with GATA motif instances that are in enhancer chromatin states in K562 cells [27], and **(B)** 1,499 DNA segments that are in enhancer chromatin states in K562 cells [28]. The assayed DNA segments were categorized as TF bound or TF unbound based on overlap with ChIP-seq peaks for EP300, TAL1, GATA1, or GATA2 in K562 cells [51]. The distribution of enhancer activities (from [27] and [28]) in each category is presented as box plots. The total numbers of DNA segments in each category are given at the bottom of the plots. The horizontal internal line and the diamond shape indicate the median and mean of enhancer activity in each category, respectively.

The primacy of TF occupancy in determining enhancer function is a robust result. We extended our analysis to include the recent results of massively parallel reporter assays (MPRAs) on 320 candidate enhancers with GATA motifs [27] and 1,499 candidate enhancers [28]. We annotated each tested candidate enhancer for occupancy by EP300, TAL1, GATA1, and/or GATA2 in K562 cells and then evaluated whether presence of TF binding associated with strength of enhancement. In both datasets, candidate enhancers that were bound by one or more of the TFs drove significantly higher levels of expression than those were not bound (*P* value = 1.5E-12 and 4.6E-05 for datasets from [27] to [28], respectively, using the Welch two sample *t*-test) (Figure 5; effects for each TF are shown in Additional file 2: Figure S7).

### Candidate regulators of TAL1-bound active enhancers

**Figure 6:**
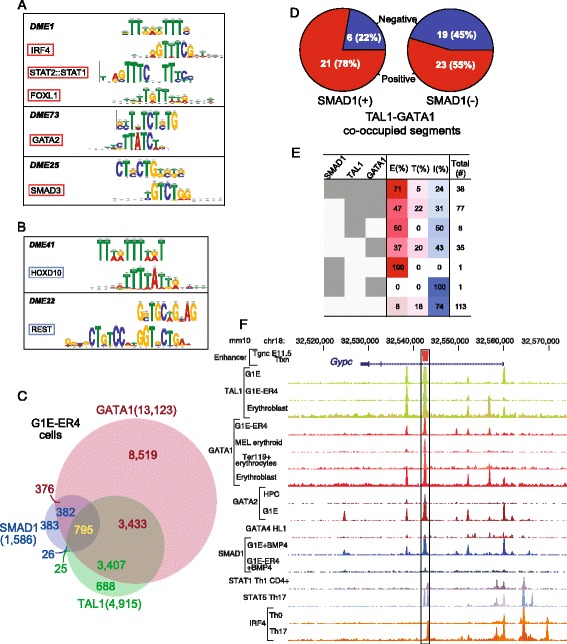
Candidate regulators of TAL1-bound active enhancers. **(A)** Motifs that distinguish TAL1 OSs that are active enhancers from those that are inactive. The motif discovered by DME2 is given on the first line of each box, followed by the known TF binding site motifs discovered by TOMTOM, all shown as aligned logos. **(B)** Motifs that distinguish TAL1 OSs that are inactive enhancers from those that are active. **(C)** Venn diagrams show genome-wide overlaps between SMAD1, TAL1, and GATA1 peaks in erythroid lineage, G1E-ER4 + E2 cells. Number of regions bound by these peaks is shown. **(D)** Power of SMAD1 binding on enhancer activity in transgenic mice. **(E)** Heat map depicting the effect of co-localization of SMAD1, TAL1, and GATA1 in different combinations on success rate *in vitro* enhancer assay (transfections into hematopoietic cell line). **(F)** Shown is an intron of the *Gypc* gene in the mouse genome, along with ChIP-seq binding profiles for TAL1 (GEO sample numbers: GSM746555-56, GSM746571-72, GSM746583_84), GATA1 (GSM453997, GSM417015, GSM1151146, GSM746581-82), GATA2 (GSM641911, GSM722387), GATA4 (GSM558904), SMAD1 (GSM722388, GSM722391), STAT1 (GSM994528), STAT5 (GSM652878), STAT5b (GSM1014575, GSM671418), IRF4 (GSM1004833_35, GSM1004821), and FOXO1 (GSM1131775, GSM998924) in hematopoietic cells, in descending order. ChIP-seq binding signals (bigwig) were obtained from CODEX [59].

We hypothesized that additional protein binding to the TAL1 OSs may contribute to their activity, which predicts that binding site motifs for such proteins would be present at significantly different frequencies in active *versus* inactive groups of TAL1 OSs. We used the Discriminating Matrix Enumerator program version 2, DME2 [55], to identify differentially enriched motifs, analyzing separately the sets of TAL1 OSs tested in each assay (Additional file 1: Figure S8A). The highest scoring DME2 motifs in each of the resulting sets of enriched motifs were used to search through two databases of known motifs, JASPAR [56] and UniPROBE [57], using the motif comparison tool TOMTOM [58] (Additional file 1: Tables S10 to S13). Further investigation of the motifs with good statistical support that were enriched only in active or inactive TAL1 OSs (Additional file 1: Table S14) revealed five particularly informative motifs from this analysis. Motifs enriched in enhancer-active TAL1 OSs matched binding site motifs for several proteins not previously implicated in enhancement by TAL1, including members of the IRF, STAT, FOX, and SMAD families of proteins (Figure 6A). Conversely, motifs that were enriched in TAL1 OSs not active as enhancers included the binding site motifs for HOXD10 and REST (Figure 6B).

For these proteins implicated in enhancer activity by motif enrichment, we searched for evidence that they were co-localizing with TAL1 and potentially played a role in enhancement. Our search was facilitated by the extensive compilation of ChIP-seq datasets in the CODEX resource [59]. Members of the SMAD family of proteins, which mediate TGF-beta- and bone morphogenic protein (BMP)-signaling pathways, were particularly attractive candidates. The receptor-regulated SMADs, including SMAD1 and SMAD3, form a trimer with a co-SMAD to regulate the expression of target genes [60], but the SMAD proteins require association with other factors to bind effectively to DNA and activate transcription [60,61]. Recent studies showed that in response to BMP4 treatment, SMAD1 tends to co-bind to DNA with the master erythroid GATA factors in G1E and induced G1E-ER4 cells [38]. By integrating these ChIP-seq data for SMAD1 occupancy [38] into our analyses, we found that SMAD1 occupancy overlaps extensively with not only GATA1 but also TAL1 occupancy (Figure 6C). Seventy-six percent of SMAD1 OSs overlap with either GATA1 OSs or TAL1 OSs, and 795 sites were bound by all three proteins. An example of co-occupancy is shown in Figure 6F. A similarly large overlap between SMAD1 peaks (55%) and TAL1 OSs or GATA1 OSs was seen in human K562 cells (Additional file 2: Figure S9A). Functional term enrichment analysis by GREAT (Genomic Regions Enrichment of Annotation Tool; [62]) showed that the presumptive gene targets of SMAD1 in induced G1E-ER4 cells are enriched for hematopoietic activities, including heme biosynthetic process, regulation of myeloid cell differentiation, erythropoiesis, erythrocyte morphology, and erythrocyte cell number (Additional file 2: Figure S10A and S10B).

Inclusion of SMAD1 binding with TAL1 occupancy improved the accuracy of enhancer prediction. All the TAL1 OSs that were co-bound by SMAD1 in G1E-ER4 cells were also bound by GATA1 (Figure 6C), and thus we evaluated TAL1-GATA1 co-occupied DNA segments for enhancer activity with or without SMAD1. Of the 27 TAL1-GATA1-SMAD1 co-bound regions tested for enhancer activities in mouse transgenic assays, 21 (78%) showed reproducible *in vivo* enhancer activity in different tissues (Figure 6D; Additional file 1: Table S15). In contrast, of the 42 TAL1-GATA1 co-bound segments that were not bound by SMAD1, a smaller percentage (55%) were active in this assay (Figure 6D). A similar positive effect of SMAD1 occupancy along with TAL1 and GATA1 co-occupancy on accuracy of enhancer predictions tested in transgenic mice was observed when ChIP-seq data from human K562 cells was used to identify candidate enhancers (Additional file 2: Figure S9B). The presence or absence of SMAD1 had little effect on the tissue distribution of enhancer activity of TAL1 and GATA1 co-occupied DNA segments (Additional file 2: Figure S11A and S11B).

The positive effect of including SMAD1 in enhancer predictions was also observed in results from transient transfections of hematopoietic cells. Inclusion of SMAD1 binding along with TAL1 and GATA1 binding produced a success rate of 71% in our transfection assays (Figure 6E, Additional file 1: Table S6). Moreover, partitioning the expression results of 1,499 candidate enhancers tested in a massively parallel reporter assay [28] by TF occupancy in K562 cells showed that DNA segments bound by TAL1 and SMAD1 gave a higher distribution of expression levels than those not bound by the two proteins (Additional file 2: Figure S9C). Of the 30 SMAD1 peaks that overlap with active enhancers bound by TAL1, the DNA sequences of 7 (23%) have matches to the SMAD motif (Additional file 1: Table S16), suggesting that in these cases the recruitment of SMAD1 involved direct binding to its preferred binding site in DNA, which is consistent with the motif enrichment.

The other proteins whose binding site motifs were enriched in enhancer-active TAL1 OSs also overlapped with TAL1 OSs but much less frequently than seen for SMAD1 (Additional file 2: Figure S13). ChIP-seq binding profiles for IRF2 have been determined in human proerythroblasts [63], and 3% and 6% of these overlap with TAL1 peaks determined in human proerythroblasts [64] and K562 cells [51], respectively. Binding profiles have been determined for the candidate proteins in many non-erythroid cell types, and we present results on overlaps with representatives of the other protein families. STAT1 binding profiles have been determined in mouse macrophages [65] and T cells [66]. Despite the fact that these are not erythroid cells, in both cases, about 1% of the STAT1 peaks overlap with TAL1 OSs determined in G1E-ER4 cells. Likewise, about 2% of the binding sites for FOXO1 in mouse CD4+ cells [67] and SMAD3 in mouse B cells [68] overlapped with TAL1 OSs in G1E-ER4 cells. Examples of the binding signals for many of these proteins are shown for the *Gypc* gene (Figure 6F for mouse; Additional file 2: Figure S12 for human). In each of these sets of TAL1 OSs that are also bound by IRF2, STAT1, FOXO1, or SMAD3, a small number have been tested for enhancer activity in transgenic mice, transfected cells, or both. In all cases, a substantial proportion of the tested TAL1 OSs was active, ranging from 47% to 100% (Additional file 1: Table S17).

### Inclusion of SMAD1 and CAGE tags refine enhancer prediction for higher accuracy

**Figure 7:**
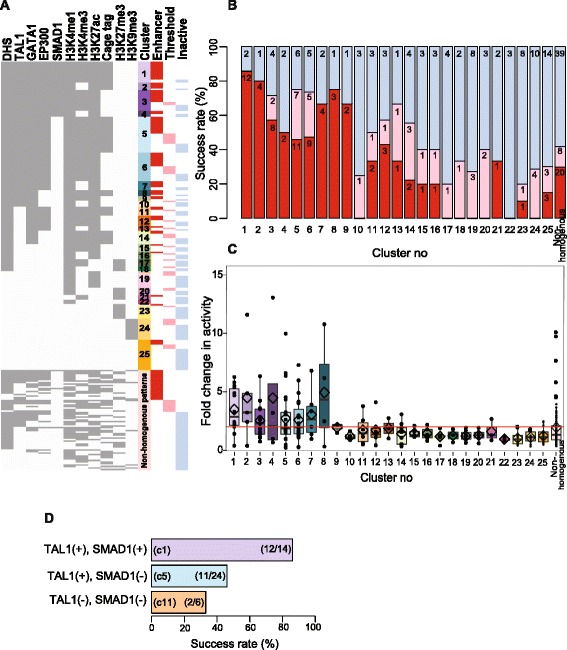
Combinations of epigenetic features and their association with enhancer activity. **(A)** Each row of the diagram shows the features and activity results for 1 of the 273 tested DNA segments. The presence or absence of the 11 epigenetic features is represented by grey and white, respectively. The tested segments were organized into clusters by DBSCAN [69] using the binary representation of the epigenetic features. Each tested DNA segment is also categorized by activity in the transient transfection assay, denoted by a colored entry in the last three rows (red = active, pink = threshold, blue = inactive). Success rates **(B)** and activities **(C)** in enhancer assays on 273 DNA segments in each cluster formed by DBSCAN. **(D)** Power of SMAD1 and TAL1 binding to identify enhancers.

The previous analysis examined the impact of feature combinations usually without regard for other features; each tested DNA segment was included as either a positive or negative prediction for each feature combination. In a complementary approach, we assigned each of the 273 tested DNA segments to only one of several discrete groups, and each group was defined by a consistent pattern of the presence of some features and absence of others. We also added SMAD1 binding data [38] based on the positive results described in the previous section. We used density-based spatial clustering of applications with noise (DBSCAN; [69]) to identify homogenous clusters while placing the less informative non-homogenous combinations into a separate category. DBSCAN revealed 25 homogenous clusters of feature combinations that contribute differentially to the response of enhancer activity (Figure 7; Additional file 1: Table S6). Each cluster was then evaluated by the prevalence of three enhancer activity categories (active, threshold, inactive) and their median fold change in activity in the enhancer assay.

Our analysis of the enhancer activity within these discrete groups of DNA segments not only shows the importance of TF binding as a predictor of enhancers but also reveals highly accurate combinations. Nine features associated with transcriptional activity can be considered positive: DHS, TAL1, GATA1, EP300, SMAD1, H3K4me1, H3K4me3, H3K27ac, and CAGE tags. The other two, H3K27me3 and H3K9me3, can be considered negative. The positive features tended to occur together, and these displayed a high frequency of active enhancers (Figure 7A). Cluster 1, containing all the positive features, and cluster 2, lacking only the promoter-associated mark H3K4me3, both had remarkably high frequencies of active enhancers, that is, 86% (12 out of 14) and 80% (4 out of 5), respectively (Figure 7A,B). The median fold changes in activity were also strikingly high, 2.9-fold and 3.2-fold, respectively (Figure 7C). Clusters 3 to 7 lacked SMAD1 and/or CAGE tags, and while they had high frequencies of active DNA segments (46% to 67%), they were substantially lower than clusters 1 and 2; their median fold change was also lower. Clusters 10 to 14 exclude one or more additional TFs but retain the positive histone modifications and DHS, and these have lower frequencies of active enhancers and lower median fold changes. Thus, the presence of TFs had a strong positive effect, and the combination of all four TFs along with positive histone modifications, CAGE tags, and DHSs gave the highest frequency of accurate enhancer predictions. The combination of TAL1 plus SMAD1 plus CAGE tags (which always co-occurred with DHS, GATA1, and EP300) was a particularly strong predictor of enhancement (Figure 7C).

In contrast, DNA segments with positive histone modifications and DHS but no TF occupancy (clusters 15, 16, 19, and 22) were rarely active as enhancers. The median enhancer activity of the DNA segments ranged from 0.9 to 1.3 (Figure 7C), and the frequency of enhancers ranged from 0% to 20% (Figure 7B). As expected, DNA segments with none of the ten features (cluster 25) or with only negative histone modifications (clusters 23 and 24) were rarely active in the enhancer assay.

### Preservation of binding site motifs is a positive predictor of enhancement

The overall level of sequence conservation surrounding the TAL1 OSs, based on interspecies comparisons, was not strongly associated with activity in either enhancer assay. The large majority of both sets of TAL1 OSs, tested in transient transfections or in transgenic mice, had quite low levels of conservation aggregated across the bound segments (Additional file 2: Figure S14AA; Additional file 1: Table S18). The level of conservation, estimated by the phastCons score [70] and phyloP [71], had weakly positive associations with level of activity in the transient transfection assay (correlation coefficients from linear regression *R* = +0.173 and *R* = +0.015, respectively, Additional file 2: Figure S14B).

We expected that evolutionary constraint would be most intense on the protein binding sites, and thus we focused on preservation of a TF binding site motif between mouse and human to monitor more localized constraint. The GATA motif (WGATAR) is the major sequence determinant of GATA1 binding, and it is also the TF binding site motif most strongly associated with TAL1 occupancy, reflecting a strong role of GATA factors in directing the binding of TAL1 [37,72]. Individual instances of this motif in the TF-bound sites tested in enhancer assays were categorized as having (a) a GATA motif preserved between mouse and human, (b) a GATA motif in mouse but not human, or (c) no GATA motif in mouse (Additional file 2: Figure S15A and B). Each TF OS was then assigned hierarchically to categories a to c, so that a DNA segment with both preserved and lineage-specific motifs was placed in the preserved category (Additional file 1: Table S19). Evaluation of the enhancer activity for the TF OSs in each motif-conservation category revealed a positive association with preservation of the GATA binding site motif. The distribution of enhancer activities was significantly higher for DNA segments with a preserved motif than for those in which the motif is not preserved; this is the case both for 151 DNA segments bound by GATA1 (*P* value = 0.017) and for 115 DNA segments bound by both GATA1 and TAL1 (*P* value = 0.038) (Additional file 2: Figure S15B). Furthermore, the frequency of observing enhancer activity was higher for the TF-bound DNA segments with the preserved GATA motif (60% to 65% compared to 41% to 44% for non-preserved motifs in the GATA1-bound and GATA1-TAL1 co-bound DNA segments, respectively).

## Discussion

### Importance of TF binding for accurate prediction of enhancers

This study gives important insights into the relative contributions of different epigenetic features to the identification of active enhancers. Our examination of TAL1 OSs showed that predicting enhancers based on occupancy by a single critical TF was quite accurate, giving a success rate of 56% in transient transfections of hematopoietic cells and 65% in transient transgenic mice. Moreover, our meta-analysis of 273 tested DNA segments showed that TAL1 binding was as accurate for the prediction of enhancers as binding by the coactivator EP300. Binding by either factor was the single feature with the greatest predictive power. Previous studies used other tissue-specific transcription factors, GATA1 binding in erythroid cells [24] and MYOD binding in muscle cells [25], to predict enhancers but with lower success rates (confirmed for GATA1 in our meta-analysis). Thus, binding profiles for some TFs appear to be better predictors than others, which raises the issue of what properties of TFs make them effective as predictors of enhancers. A recent study of gene activation by NOTCH1 complexes in T cells found that the dynamically occupied DNA segments are involved in regulation [73]. One interpretation of our results is that TFs displaying a highly dynamic pattern of binding across differentiation, such as TAL1 during hematopoiesis [37], may be the better predictors of active enhancers. The increased accuracy of enhancer prediction when TAL1 OSs are combined with SMAD1 occupancy may also reflect the effectiveness of dynamic binding as a predictor. After activation by BMP signaling, SMAD1 co-binds with lineage-specific master regulators such as GATA1 [38] and TAL1 (this report). This binding by SMAD1 suggests a high accessibility of the enhancer-active TAL1 OSs to binding by additional transcriptional activators, which is a property expected for dynamically active TF-bound sites. The TAL1-SMAD1 co-bound DNA segments were also marked by CAGE tags (Figure 7A); this signature of transcription could also reflect a dynamic state at the active enhancers.

The DNA segments bound by the TFs examined were almost uniformly in chromatin with histone modifications associated with activity [37,41], and thus including positive epigenetic features such as H3K4me1 and H3K27ac modifications [18,22] improved the accuracy of enhancer prediction only modestly. Surprisingly, the level of methylation at H3K4 did not appear to be a major determinant of enhancer activity. Importantly, DNA segments associated with these HMs but with *no* binding by TAL1, GATA1, EP300, or SMAD1 were almost never experimentally validated as enhancers. This result suggests that many DNA segments in chromatin with HMs associated with activity are not directly or independently involved in gene regulation.

Our conclusions help explain recent results using MPRAs to evaluate the accuracy of enhancers predicted by HMM-based integrations of histone modification signals [26] in human cell lines. Kwasnieski *et al*. [28] sampled 1,800 candidate enhancers predicted by HMM-based chromatin states in K562 or H1 embryonic stem cells and found that 11% and 26% of the predicted strong and weak enhancers, respectively, were active in a reporter gene transfection assay in K562 cells. Kheradpour *et al*. [27] used the chromHMM strong enhancer predictions but restricted their analysis to over 2,000 with matches to binding site motifs for cell-type specific transcription factors. For candidate enhancers with motifs indicative of binding by transcriptional activators in K562 and HepG2 cells, they estimated that 42% and 26% of the candidate enhancers were active when the binding site motifs were conserved or nonconserved, respectively. The higher proportions that were active when binding site motifs were conserved are consistent with our conclusion that binding by a key TF is the major determinant of enhancer activity. Indeed, Kwasnieski *et al*. [28] found that candidate enhancers that were bound by AP1 ascertained by ChIP-seq data, [51] drove higher levels of expression that those are not bound, confirming on a large scale the results of early studies [74,75]. Furthermore, the lower proportions active when TF occupancy was not inferred are consistent with our observation that histone modifications with TF binding excluded are poor predictors of enhancer activity. The overall success rates reported with MPRAs are consistently lower than those observed our work. Further studies are needed to better understand the factors contributing to these differences, such as sensitivities of the assays, use of very short DNA fragments in MPRAs, methods of ascertaining the predictions, and effects of sample sizes.

While many studies point to the importance of the epigenetic features studied in our work, their quantitative contributions individually and in combination are not consistently agreed upon across several recent studies [27-29]. We observe experimentally determined occupancy by TFs or coactivators to be of primary importance, and our re-analysis of the results of MPRA experiments [27,28] shows a similar positive effect for TF occupancy. Almost all active enhancers in our study are in accessible chromatin, but the vast majority of DHSs lack binding by enhancer-associated TFs, and DHSs include many ‘false positive’ predictions for enhancers. In contrast, Kwasnieski *et al*. [28] report DHS to be the strongest feature distinguishing active from inactive chromHMM enhancer predictions, while models built on TF ChIP-seq data perform less well than those built on DHS or incorporating motifs. We find that H3K27ac has good predictive power, in agreement with Erwin *et al*. [29] but Kheradpour *et al*. [27] find a local ‘dip’ in H3K27ac to be a strong predictor, while Kwasnieski *et al*. [28] find that lower levels of H3K27ac are associated with active enhancers. These differences may be more apparent than real, and we predict that further experiments and consistent analyses with common criteria for evaluating predictive power will reveal more agreement among results. While the contributions of the various epigenetic features have not yet been definitively determined, the primary importance of TF binding is a robust result.

Motif enrichment analyses coupled with comparisons to genome-wide binding profiles identified additional proteins that may co-bind with TAL1 and contribute to enhancement during erythropoiesis, specifically SMAD proteins, interferon regulatory factors (IRFs), the IRF partners STAT2::STAT1, and forkhead box (FOX) family proteins. The SMAD1 occupancy pattern [38] overlaps substantially with that of TAL1 and GATA1 in both mouse and human erythroid cells, and we showed that DNA segments bound by all three TFs were active enhancers at very high rates (86% for transient transfection and 78% for transgenic mice, which are comparable to the highest success frequencies reported for predicted mammalian enhancers [1,7]. Previous studies of binding motifs [76], ChIP-seq binding profiles [38], and protein interactions [77] had implicated SMAD binding along with lineage-specific TFs in control of erythroid regulation, and our analyses support a strong role for the SMADs in TAL1-mediated enhancement. A similar process has been proposed in which neuronal genes are activated by the recruitment of SMAD1-EP300 by neurogenin [78]; both neurogenin and TAL1 are bHLH TFs. About one-quarter of SMAD1-TAL1 co-bound active enhancers have SMAD binding site motif instances in the DNA sequence, suggesting recruitment by direct binding of SMAD1. However, in these and the remaining cases of SMAD1 binding, a role for protein-protein interactions in recruitment is also plausible. The involvement of IRFs in TAL1-mediated enhancement is supported by previous studies implicating the family of IRFs in determining binding by GATA1 [40] and erythroid enhancement [63]. Furthermore, exploring the FactorBook of ENCODE ChIP-seq data [79] showed that IRF1-bound sites in human K562 cells were enriched for co-binding by TAL1, GATA2, and GATA1. Other studies have also implicated STAT proteins [80-82] and FOX proteins [83-86] in erythropoiesis, as suggested by our motif analysis. Genome-wide binding profiles for members of these families have not yet been determined in erythroid cells, and such datasets should be considered as a high priority for future studies.

Most of the DNA segments bound by TAL1 have not been strongly conserved since the divergence of rodents and primates, just like many other DNA segments associated with regulation [10,46,87-89]. However, when we focus on the most informative transcription factor binding motifs within these occupied DNA segments, we find substantial preservation of the motif across mammalian evolution in the active enhancers. This lends further support to the conclusion that evolutionary constraint on enhancement activity leads to preservation of the binding site motif [24]. The efficacy of preservation of TF binding site motifs over large evolutionary distances was demonstrated on a very large scale in mammals in a recent MPRA study [27].

Insights from this study can be applied widely in enhancer predictions. An initial survey of the regulatory landscape can be made with high sensitivity using DHS, or an integrative modeling of histone modifications, or both. However, this large collection of DNA segments in accessible chromatin with enhancer-associated chromatin states contains many false positives for enhancer predictions. Attention should be focused on the subset that has strong evidence of occupancy by key TFs. For some cell types, a master regulator is present at many of the enhancers, such as TAL1 in erythroid cells, and ChIP-seq data for that regulator will greatly improve the specificity of enhancer predictions. Further restriction to a dynamically active subset, such as those bound even by a different TF in response to a stimulus (for example, SMADs in response to BMP signaling) and/or with evidence of transcription (CAGE tags), may enrich for active enhancers. A more broadly applicable feature is occupancy by the general coactivator EP300. Previous studies have shown that it is an accurate predictor of enhancers [7], and our study shows that it performs as well as the cell type-specific TF TAL1 in erythroid cells. Thus, ascertainment of EP300 in many cell types should be a high priority for better prediction of enhancers broadly. If experimentally determined binding data for relevant TFs are not available, matches to TF binding site motifs can be used. In this case, preference should be given to the ones that are preserved over a broad evolutionary span [24,27].

### Potential functions for enhancer false positives

The accuracy of enhancer prediction described in this work is high, and for favorable combinations of features, it is similar to some of the most effective predictors used previously [7,8]. While this result is encouraging, the fact remains that substantial fraction of predictions did not show activity in the assays. These predicted CRMs, active or inactive, possess a striking array of features strongly associated with activity, such as TF occupancy, positive histone modifications, and DNase accessibility. The success rates for the CRM predictions are similar for both the transient transfection and the transgenic mouse assays. Thus, it is unlikely that one assay is seriously under- or over-counting the biologically meaningful enhancers. Rather, we suggest that other functional roles could be played by the predicted CRMs that were inactive in the enhancer assays. These other roles could include enhancement in tissues or at developmental stages not examined here. Future work with high throughput assays [90-92] and activity-based selections [93-95] should interrogate a broader set of lineages and conditions. Also, the predicted CRMs could have roles in negative regulation [96]. The motif enrichment supports this hypothesis. One of the motifs significantly enriched in the TAL1 OSs that were not active as enhancers matches the binding site motif for REST (NRSF). This is a known repressor, and thus it is possible that co-occupancy of REST with TAL1 may lead to down-regulation of expression. The expression profile for NRSF in BioGPS [97] reveals strong expression in early erythroblasts, supporting a potential role for this repressor during erythropoiesis.

### Interpreting the results of reporter assays

Both types of assays conducted in this study remove DNA sequences from their chromosomal location and place them close to a promoter and reporter gene, which is then tested for expression either as a partially chromatinized unintegrated plasmid (transient transfections) or after random integration into a chromosome (transgenic mouse). Thus, the natural genomic and chromosomal context is lost, and this must be kept in mind in interpreting the results. The reporter assays used here do provide valuable information about the activity of the tested DNA segments in mechanistic aspects of gene regulation, such as having a positive effect on expression and thus an implication in enhancement. An understanding of the full biological role of each DNA segment in its natural context will require genome editing approaches followed by deep phenotyping. With current technologies, such studies examine small numbers of regulatory regions (albeit with profound insights). Selection of targets for genome editing can be improved both by increasing the accuracy of enhancer predictions and by generating empirical evidence of activity in the higher throughput (but context independent) assays. The data and analyses reported here contribute to both these activities, and they should be considered as part of a larger effort to build genome-wide models of gene regulation that incorporate information about chromosomal position and cell context.

### Activity of erythroid-ascertained enhancers in brain, heart, and other tissues

Despite the fact that TAL1 occupancy and other epigenetic features were ascertained in erythroid cells, the TAL1-bound enhancers that were positive in transgenic mouse embryos were active in brain, heart, and other tissues. The time at which the transgenic embryos were examined (day 11.5) is earlier than the time of highly active hematopoiesis in the mouse fetal liver (day 14.5 and later; [98,99]), which explains the infrequent observation of fetal liver enhancement. Explanations for expression in other tissues include (a) activity of TFs present in erythroid cells that are also present in other tissues, (b) activity of paralogous proteins in other tissues that are related to the erythroid TFs and bind to the same binding site motif, and (c) pleiotropic enhancers normally active in multiple tissues. These possibilities are not mutually exclusive. For example, expression in the brain could reflect all three, since (a) TAL1 is also present in the brain, and (b) it co-operates with its paralog TAL2 and GATA2 during GABAnergic neurogenesis in the midbrain [100]. We recently showed that DNA segments at which TF occupancy is conserved from mouse to human tend to be active in multiple tissues, including examples of activity in both erythroid cells and brain (possibility c, [46]).

In addition to pleiotropic enhancers, the ability of TAL1 OSs to enhance expression in the heart could reflect a role in fetal cardiac endothelial cells in hematopoiesis. Definitive hematopoietic stem cells are derived from a subset of endothelial cells along the fetal dorsal aorta in the aorta-gonad-mesonephrous region (AGM), and they migrate to the fetal liver to establish high-level hematopoiesis [99,101]. Recent studies indicate a close connection between early cardiac development and hematopoiesis. Early cardiac progenitors express hematopoietic transcription factors, such as TAL1 and GATA1 [102], and Nakano *et al*. [103] demonstrated the hemogenic potential of the heart tube endocardium. Furthermore, TAL1 binding to primed enhancers in multipotential cardiovascular mesoderm can repress cardiac fate and sustain hematopoietic fate [104]. Thus, some of the TAL1 OSs that are active as enhancers in the transgenic fetal heart could represent enhancers that are normally active in hemogenic cells within the developing heart. A possible example is a TAL1 OS (peak ID: 1845 in Additional file 1: Table S1) that corresponds to a mouse *Runx1* intronic enhancer active in hemogenic endothelial cells and hematopoietic stem cells [105]. Further studies of epigenetic features in specific tissues at day 11.5 of mouse development will help elucidate the landscape of enhancers and their dynamics during development [106].

## Conclusions

TAL1 occupancy in erythroid cells was an accurate predictor of enhancer activity, and the accuracy was improved by including binding by SMAD1 and evidence of transcription (CAGE tags). Among epigenetic features commonly used for enhancer prediction in mammalian cells, binding by key TFs was more accurate than histone modification patterns or chromatin accessibility.

## Methods

### ChIP-seq data for epigenetic features

ChIP-seq datasets for multiple epigenetic features [10,36,38], numbers of peaks, and filenames for downloads are given Table 1.

### Data access

ChIP-seq data are deposited in GEO as the Series GSE51338 and GSE61349, and they are available from the Mouse ENCODE portal (http://mouse.encodedcc.org) and the ENCODE portal (https://www.encodeproject.org).

### Enhancer assays by transient transfection for K562 cells

Seventy DNA segments occupied by TAL1 in G1E-ER4 cells treated for 24 h with estradiol were amplified from mouse DNA and cloned into a plasmid vector with the firefly luciferase reporter gene driven by the *HBG1* promoter (Additional file 2: Figure S2A; Additional file 1: Table S2; [12]). The test constructs were transiently transfected into K562 cells in a 96-well plate using 0.14 μg of plasmid DNA containing firefly luciferase reporter and 0.00035 μg of co-transfection control plasmid expressing *Renilla* luciferase in OptiMEM medium, adding 0.14 μl of PLUS Reagent and 0.21 μl Lipofectamine LTX per well. The cells were plated at 2.8 × 10^4^ cells per well. Each plasmid was transfected in quadruplicate wells for each experiment, and each lysate after transfection was measured twice, for eight technical replicates. Each plasmid was tested in at least two separate experiments (biological replicates with transfections on different days).

Cell extracts were harvested 48 h after the transfection, and firefly and *Renilla* luciferase activities were measured in Promega’s dual luciferase assay. For each of quadruplicate transfections, at least two measurements were made on the cell lysates for a total of eight measurements of both firefly and *Renilla* luciferases for each construct in each experiment. The ratio of firefly luciferase activity of the test DNA to the *Renilla* luciferase activity of the co-transfection control was normalized by the ratio of firefly luciferase activity from the parental vector to the *Renilla* luciferase activity of the co-transfection control to get a fold change. The tested fragments that have at least a twofold increase in activity are considered as active enhancers (Additional file 1: Table S3).

### Enhancer assays in transgenic mice at embryonic day 11.5

The set of TAL1 OSs were evaluated for enhancer activities in mouse transgenic assays by mining data in the VISTA Enhancer Browser (http://enhancer.lbl.gov/; [52]). In transgenic mouse assays, candidate DNA segments are cloned into an *Hsp68*-promoter-LacZ reporter vector. The embryos are generated and evaluated for reproducible LacZ activity at embryonic stage E11.5 day. The predicted regions showing reproducible expression in the same tissue in at least three independent transgenic mouse embryos were defined as positive enhancers. DNA segments tested in transgenic mice (examining both mouse DNA segments and the human orthologs of mouse TAL1 OSs) that overlapped with a TAL1 OS for at least 50% of the TAL1 OS were included in the study (Additional file 1: Table S4).

### Identification of significantly enriched motifs by employing the computer program, discriminating matrix enumerator

A computational method called Discriminating Matrix Enumerator (DME2, beta version 2008_08_30) [55] was used to identify overrepresented motifs of size 10 (described by scoring matrices) in TAL1 bound enhancers and non-enhancers identified by two enhancer assays: (1) ‘enhancers’ and ‘inactive’ regions determined by enhancer assay in transiently transfected K562 cells; (2) ‘positive’ and ‘negative’ regions identified by enhancer assay in transgenic mice. Before DME2 was run, G + C contents of 4 datasets (enhancer, inactive, positive, negative) produced by each enhancer assay were checked. G + C contents (%) were similar between foreground and background sets so that DME2 could be run (Additional file 2: Figure S8B). DME2 software discovered the relative enrichment of position weight matrices in the foreground set checked against the background (Additional file 1: Tables S10 to S13). Discriminatory power of each enriched motif was evaluated by the relative enrichment score given by DME2 [55]. From the 200 motifs in each set (Additional file 1: Tables S10 to S13), ten highest scored enriched DME motifs (Additional file 2: Figure S8C) were analyzed further for matches to known binding sites for mammalian transcription factors in the motif databases, JASPAR Vertebrates, and UniPROBE Mouse via motif comparison tool, TOMTOM [56-58]. One-hundred eight known motifs aligned by TOMTOM (*E* value of <1) with good statistical were analyzed further. 56 motifs enriched in both the enhancer and non-enhancer sets were removed. After filtering the aligned motifs from the output of TOMTOM, transcription factor binding sites for different families of proteins enriched in only one category remained (Additional file 1: Table S14).

We also searched for occurrences of the SMAD binding site motif in the 30 SMAD1 peaks that overlapped with active enhancers bound by TAL1. We used the FIMO tool (Find Individual Motif Occurrences; MEME version 4.10.0) run at a *P* value threshold of 0.001, with default parameters, to identify matches to the weight matrix that describes the SMAD motif overrepresented at TAL1-bound active enhancers. This set of active enhancers includes those discovered by the transgenic mouse assay and the transient transfection assay.

### Clustering algorithms

The ChIP-seq signal strength of H3K4me1, H3K4me3, TAL1, and GATA1 occupancy was calculated on each TAL1 OS in G1E-ER4 cells treated with estradiol for 24 h (Figure 2A). The TAL1 OSs were clustered into eight categories by k-means clustering (center-based) [107] based on the log_2_ transformed ratio of H3K4me1 to H3K4me3 levels, the TAL1 occupancy levels, and the GATA1 occupancy levels (Additional file 1: Table S5). Clustering at higher values for k did not bring out additional distinctive groups. Clustering was repeated 100 times, and only those OSs that could be placed in the same clusters for at least 50 times were retained for the subsequent assays and display (4,648 TAL1 OSs). In each iteration of clustering, the identity of a cluster was determined by the rank of its median of H3K4me1/H3K4me3 ratios among all clusters. The clustering was displayed by heat maps (Figure 2A). The coloring of the first column was set so that the intervals with zero value (meaning the H3K4me1/H3K4me3 ratio equals to one) were in black. The coloring of the third column was set so that the intervals with value 0.11 were in pink. This value was determined so that most (over 90%) intervals with larger values were called as GATA1 OSs, and most (over 90%) intervals with smaller values were not called as GATA1 OSs.

In addition, the 273 DNA segments in the meta-analysis were grouped by similarity in patterns of the presence or the absence of ten epigenetic features that contribute differentially to the response to enhancer activity using DBSCAN (density-based spatial clustering of applications with noise) (Additional file 1: Table S6; [69]). Twenty-five homogenous clusters with the non-homogenous DNA segments were displayed as a heat map (Figure 7). Presence of epigenetic features in the DNA segments is shown grey in color, and absence of the features is represented by white color. We set the DBSCAN parameters: the size of the ε-neighborhood of a DNA segment, Eps = 1, and the minimum number of DNA segments showing homogenous pattern of epigenetic features required to form a cluster, minpts = 3. Homogenous clusters formed by DBSCAN were numbered from 1 to 25 and colored by different colors. Non-homogenous patterns of DNA segments were put together in the lower part of the heat map.

### Measuring discriminatory power of transcription factors and histone modifications to identify enhancers

The discriminatory power of each positive feature and different combinations of them were evaluated by their sensitivity (recall) and specificity values (Additional file 1: Tables S8 and S9). The sensitivity is defined as the fraction of the DNA segments with set of features that are active enhancers. The specificity determines the fraction of the DNA segments without set of features that are inactive.

Recent results of massively parallel reporter assays used in this study were taken from two sources: Kheradpour *et al*. [27] and Kwasnieski *et al*. [28]. The 320 DNA segments predicted by GATA motif instances within enhancer chromHMM states [27] and 1,499 candidate enhancers predicted by ENCODE DNA segments by histone modifications [28] were utilized to assess the accuracy of enhancers in the presence of TF binding (all from K562 cell line). ChIP-seq Uniform Peaks for EP300, TAL1, GATA1, and GATA2 in K562 cells from ENCODE (hg19 database) were used to annotate each tested candidate enhancer for occupancy by these TFs. Out of 320 DNA segments, 247, 260, 119, and 242 [27] were bound by EP300, TAL1, GATA1, and GATA2 in K562 cells, respectively, while only 149, 154, 18 and 129 out of 1,499 DNA segments [28] were found to be bound by EP300, TAL1, GATA1, and GATA2 in K562 cells, respectively. The association between the presence of merged TF binding and strength of enhancement for both datasets was shown in Figure 5 (effects for individual TF are also shown in Additional file 2: Figure S7).

### Analyses of sequence conservation and motif preservation

PhastCons [70] and PhyloP [71] conservation score of each tested TAL1 peak were obtained from conservation tracks of UCSC genome database. PhastCons scores show the level of conservation of each nucleotide of a conserved element in a multispecies alignment (on a scale of 0, not conserved; to 1, fully conserved). PhyloP score is a measurement of the conservation or divergence of a specific alignment position (high positive values point out purifying selection while negative scores indicate acceleration). We calculated the average score for each tested TAL1 peak that is centered on the middle of the called peak and extending 50 bp on each side (Additional file 1: Table S18).

The CladiMo software package [108] was used to find all WGATAR motif instances in alignments of multiple mammalian genome sequences. Each DNA segment was categorized as having (1) a GATA motif preserved between mouse and human, (2) a GATA1 motif in mouse, not human, or (3) no GATA motif in mouse (Additional file 1: Table S18).

## Additional files

Additional file 1:
**This file includes Tables S1 to S17.**
Additional file 2:
**This file includes Figures S1 to S15 and the legends of these figures.**

## Abbreviations

CAGE: cap analysis of gene expression
ChIP-seq: chromatin immunoprecipitation followed by massively parallel DNA sequencing
CRM: *cis*-regulatory modules
DBSCAN: density-based spatial clustering of applications with noise
DHS: DNase I hypersensitive site
DME2: Discriminating Matrix Enumerator program version 2
ENCODE: The Encyclopedia of DNA Elements
EP300: E1A binding protein p300
GATA1: GATA-binding factor 1
HM: histone modification
H3K27ac: histone H3 lysine 27 acetylation
H3K4me1: histone H3 lysine 4 mono-methylation
H3K4me3: histone H3 lysine 4 tri-methylation
H3K9me3: histone H3 lysine 9 tri-methylation
H3K27me3: histone H3 lysine 27 tri-methylation
MPRA: massively parallel reporter assay
OS: occupied DNA segments
ROC: receiver operating characteristic
Sn: sensitivity
Sp: specificity
TAL1: T-cell acute lymphocytic leukemia protein 1
TF: transcription factor
TSS: transcription start site
