# Supplementary material for: Occupancy by key transcription factors is a more accurate predictor of enhancer activity than histone modifications or chromatin accessibility
Source: Epigenetics Chromatin. 2015 Apr 23;8:16. doi: 10.1186/s13072-015-0009-5 (PMC4432502; doi:10.1186/s13072-015-0009-5)
Supplement: Additional file 2: — This file includes Figures S1 to S15 and the legends of these figures. [file 13072_2015_9_MOESM2_ESM.pptx]

## Slide 1
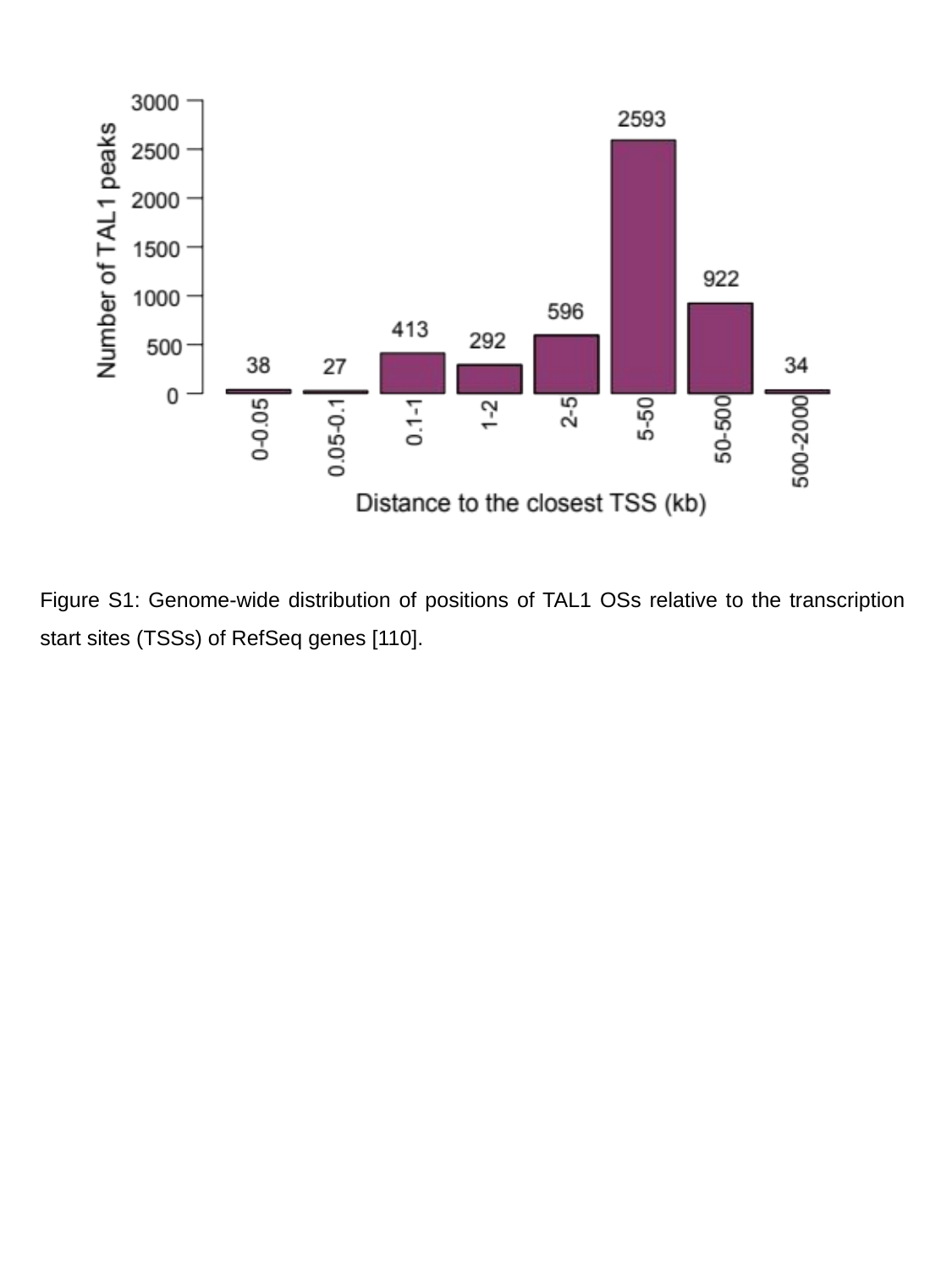

Figure S1: Genome-wide distribution of positions of TAL1 OSs relative to the transcription start sites (TSSs) of RefSeq genes [110].

## Slide 2
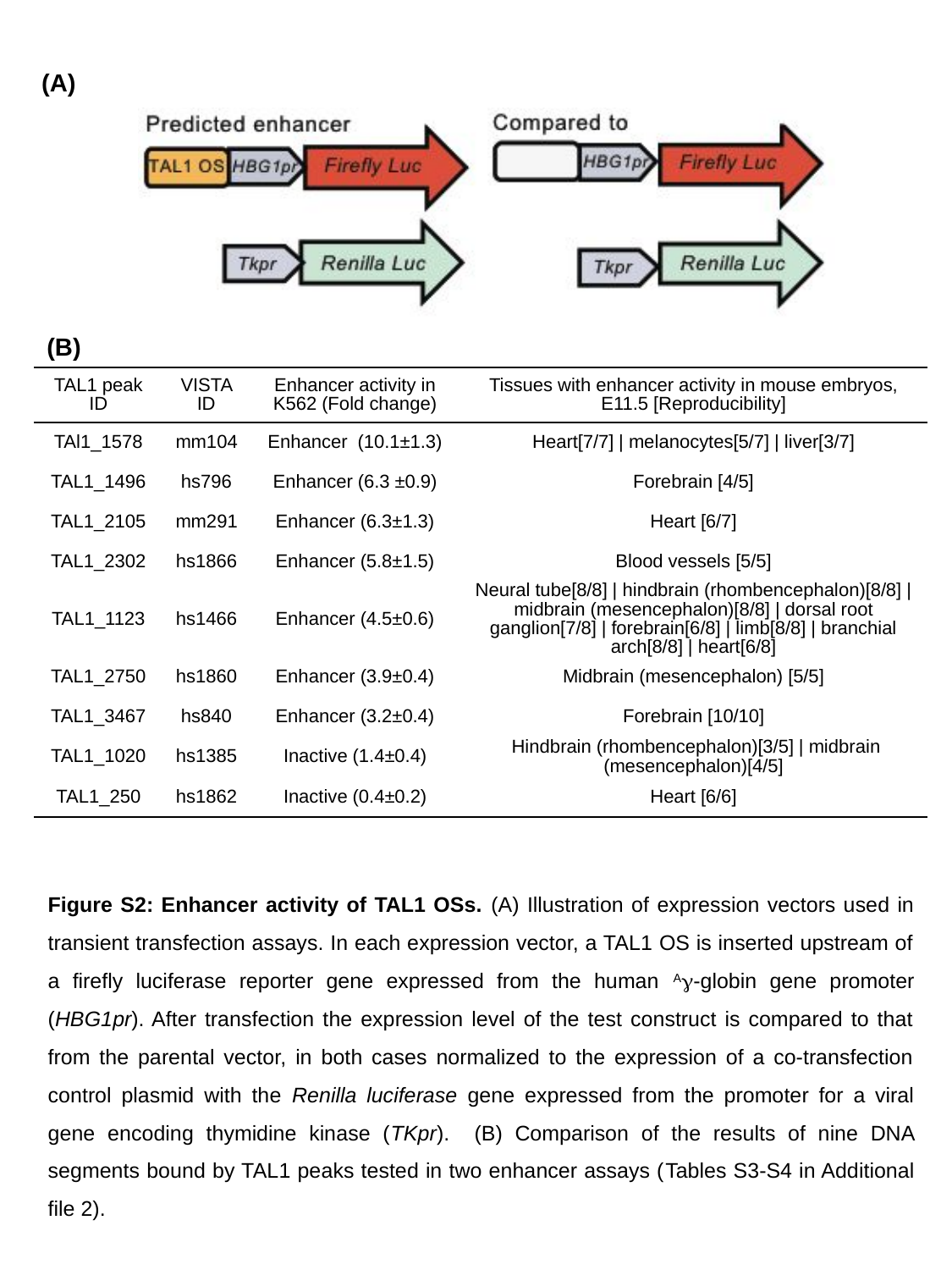

(A)
(B)
| TAL1 peak ID | VISTA ID | Enhancer activity in K562 (Fold change) | Tissues with enhancer activity in mouse embryos, E11.5 [Reproducibility] |
| --- | --- | --- | --- |
| TAl1\_1578 | mm104 | Enhancer (10.1±1.3) | Heart[7/7] | melanocytes[5/7] | liver[3/7] |
| TAL1\_1496 | hs796 | Enhancer (6.3 ±0.9) | Forebrain [4/5] |
| TAL1\_2105 | mm291 | Enhancer (6.3±1.3) | Heart [6/7] |
| TAL1\_2302 | hs1866 | Enhancer (5.8±1.5) | Blood vessels [5/5] |
| TAL1\_1123 | hs1466 | Enhancer (4.5±0.6) | Neural tube[8/8] | hindbrain (rhombencephalon)[8/8] | midbrain (mesencephalon)[8/8] | dorsal root ganglion[7/8] | forebrain[6/8] | limb[8/8] | branchial arch[8/8] | heart[6/8] |
| TAL1\_2750 | hs1860 | Enhancer (3.9±0.4) | Midbrain (mesencephalon) [5/5] |
| TAL1\_3467 | hs840 | Enhancer (3.2±0.4) | Forebrain [10/10] |
| TAL1\_1020 | hs1385 | Inactive (1.4±0.4) | Hindbrain (rhombencephalon)[3/5] | midbrain (mesencephalon)[4/5] |
| TAL1\_250 | hs1862 | Inactive (0.4±0.2) | Heart [6/6] |
Figure S2: Enhancer activity of TAL1 OSs. (A) Illustration of expression vectors used in transient transfection assays. In each expression vector, a TAL1 OS is inserted upstream of a firefly luciferase reporter gene expressed from the human Aγ-globin gene promoter (HBG1pr). After transfection the expression level of the test construct is compared to that from the parental vector, in both cases normalized to the expression of a co-transfection control plasmid with the Renilla luciferase gene expressed from the promoter for a viral gene encoding thymidine kinase (TKpr). (B) Comparison of the results of nine DNA segments bound by TAL1 peaks tested in two enhancer assays (Tables S3-S4 in Additional file 2).

## Slide 3
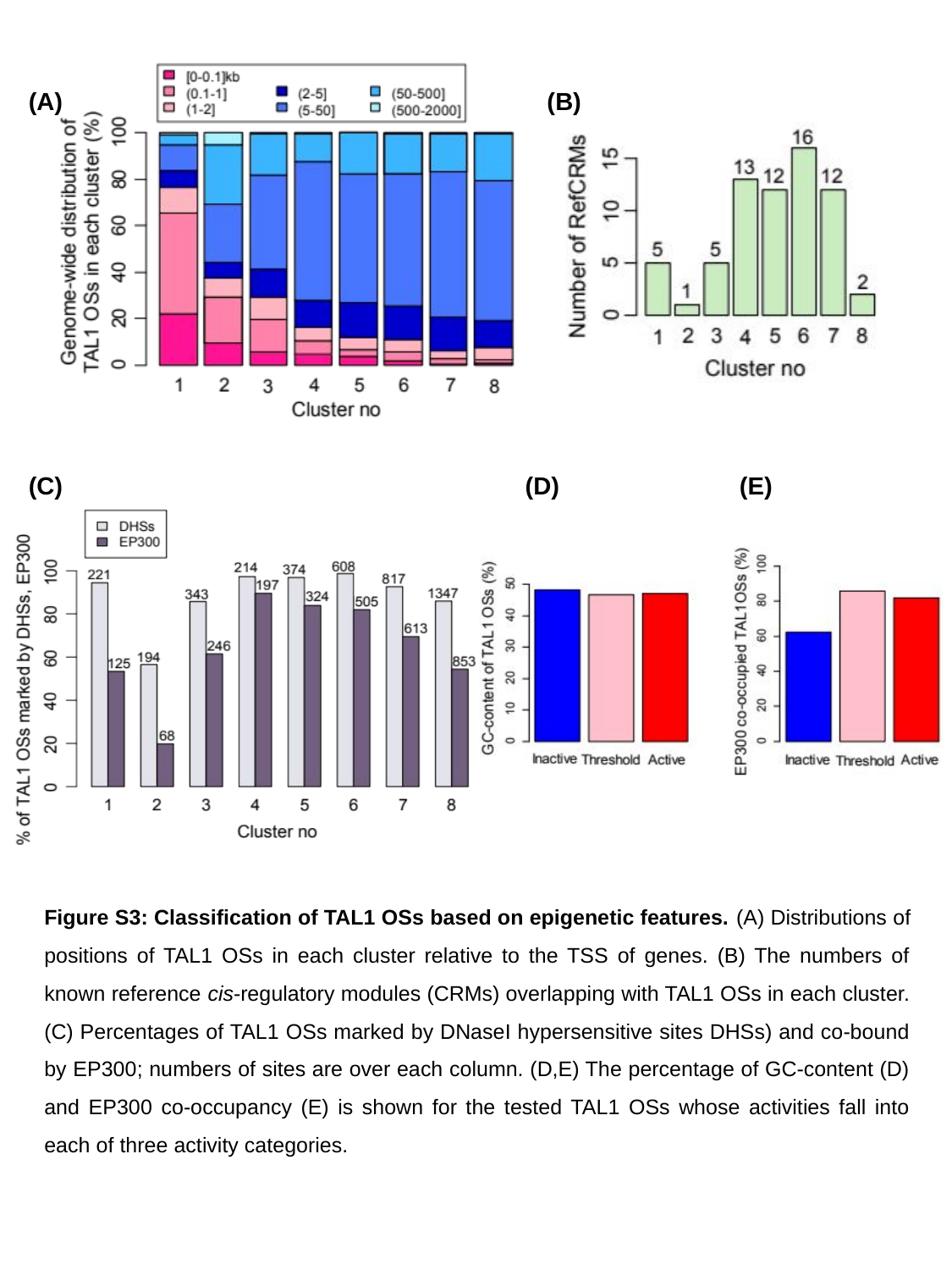

(A)
(B)
(C)
(D)
(E)
Figure S3: Classification of TAL1 OSs based on epigenetic features. (A) Distributions of positions of TAL1 OSs in each cluster relative to the TSS of genes. (B) The numbers of known reference cis-regulatory modules (CRMs) overlapping with TAL1 OSs in each cluster. (C) Percentages of TAL1 OSs marked by DNaseI hypersensitive sites DHSs) and co-bound by EP300; numbers of sites are over each column. (D,E) The percentage of GC-content (D) and EP300 co-occupancy (E) is shown for the tested TAL1 OSs whose activities fall into each of three activity categories.

## Slide 4
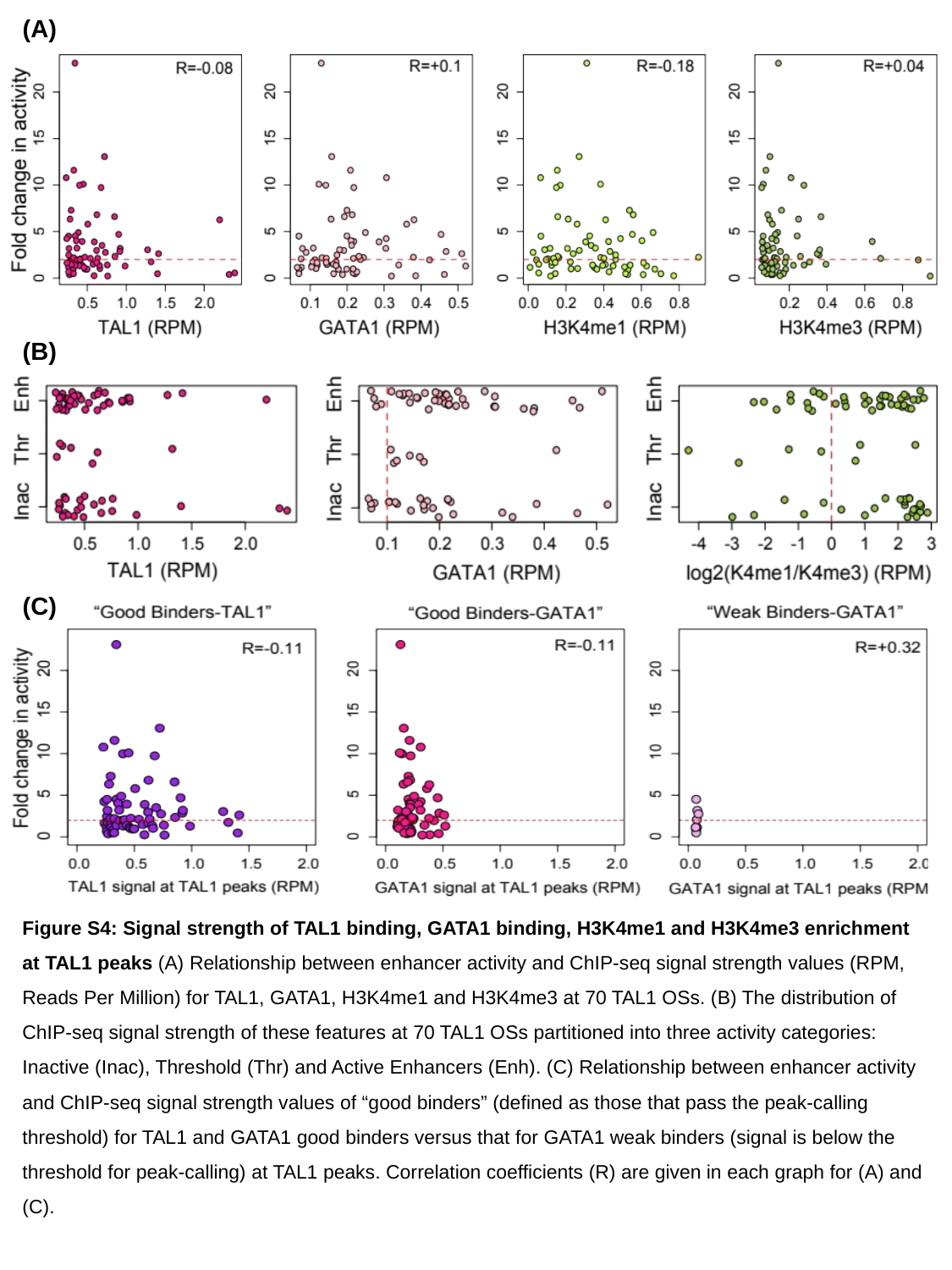

(A)
(B)
(C)
Figure S4: Signal strength of TAL1 binding, GATA1 binding, H3K4me1 and H3K4me3 enrichment at TAL1 peaks (A) Relationship between enhancer activity and ChIP-seq signal strength values (RPM, Reads Per Million) for TAL1, GATA1, H3K4me1 and H3K4me3 at 70 TAL1 OSs. (B) The distribution of ChIP-seq signal strength of these features at 70 TAL1 OSs partitioned into three activity categories: Inactive (Inac), Threshold (Thr) and Active Enhancers (Enh). (C) Relationship between enhancer activity and ChIP-seq signal strength values of “good binders” (defined as those that pass the peak-calling threshold) for TAL1 and GATA1 good binders versus that for GATA1 weak binders (signal is below the threshold for peak-calling) at TAL1 peaks. Correlation coefficients (R) are given in each graph for (A) and (C).

## Slide 5
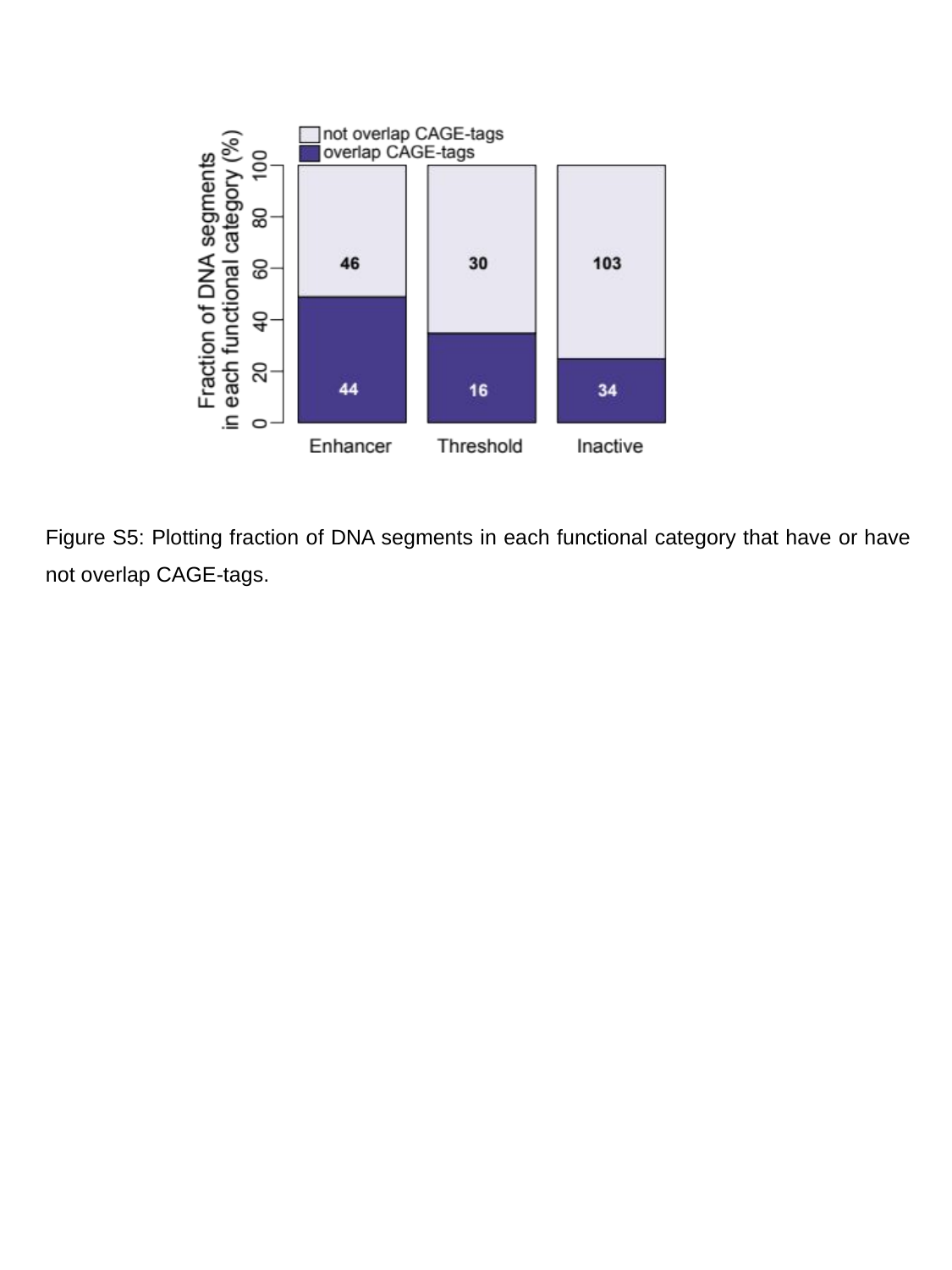

Figure S5: Plotting fraction of DNA segments in each functional category that have or have not overlap CAGE-tags.

## Slide 6
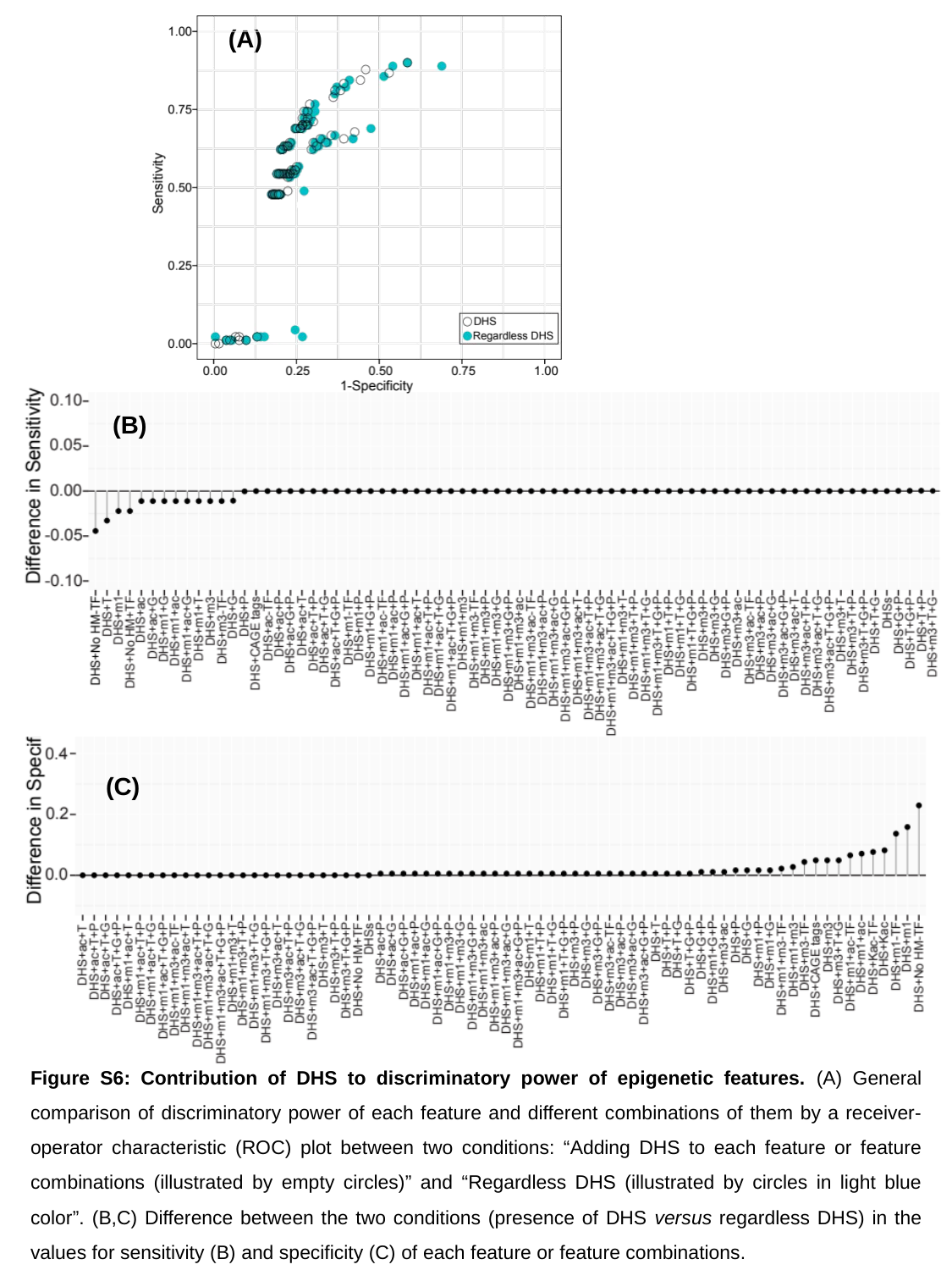

(A)
(B)
(C)
Figure S6: Contribution of DHS to discriminatory power of epigenetic features. (A) General comparison of discriminatory power of each feature and different combinations of them by a receiver-operator characteristic (ROC) plot between two conditions: “Adding DHS to each feature or feature combinations (illustrated by empty circles)” and “Regardless DHS (illustrated by circles in light blue color”. (B,C) Difference between the two conditions (presence of DHS versus regardless DHS) in the values for sensitivity (B) and specificity (C) of each feature or feature combinations.

## Slide 7
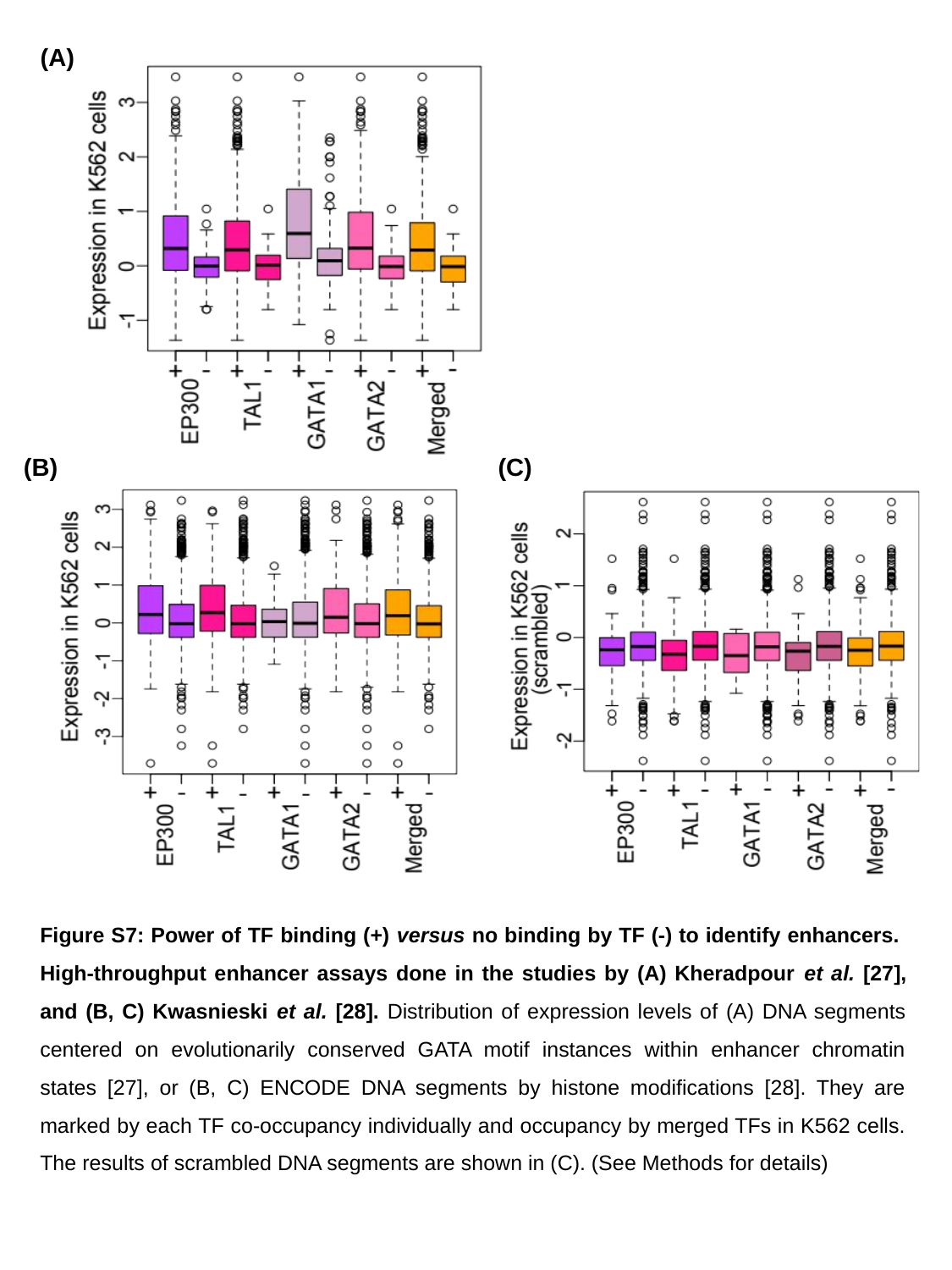

(A)
(B)
(C)
Figure S7: Power of TF binding (+) versus no binding by TF (-) to identify enhancers. High-throughput enhancer assays done in the studies by (A) Kheradpour et al. [27], and (B, C) Kwasnieski et al. [28]. Distribution of expression levels of (A) DNA segments centered on evolutionarily conserved GATA motif instances within enhancer chromatin states [27], or (B, C) ENCODE DNA segments by histone modifications [28]. They are marked by each TF co-occupancy individually and occupancy by merged TFs in K562 cells. The results of scrambled DNA segments are shown in (C). (See Methods for details)

## Slide 8
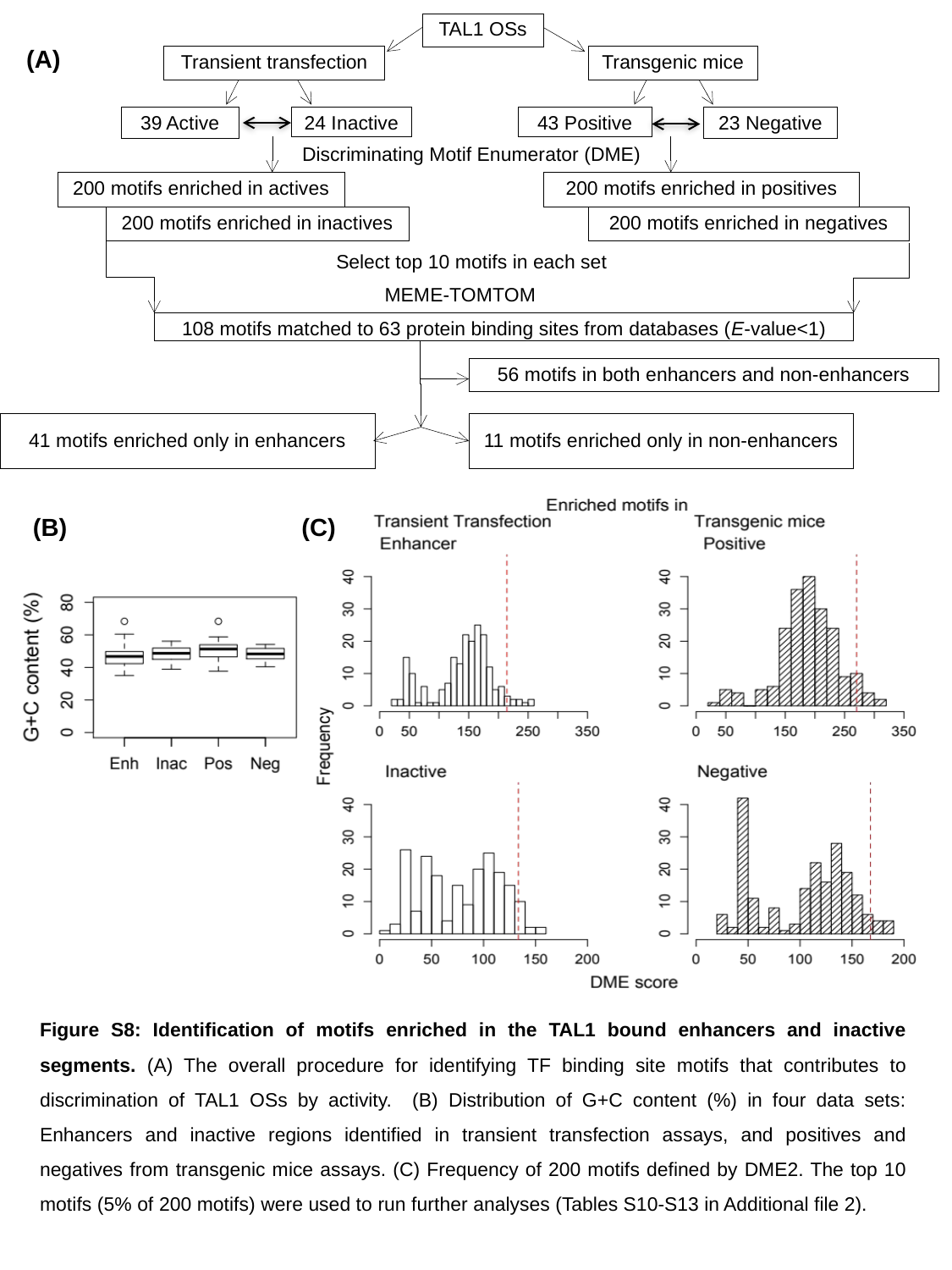

TAL1 OSs
(A)
Transient transfection
Transgenic mice
43 Positive
39 Active
24 Inactive
23 Negative
Discriminating Motif Enumerator (DME)
200 motifs enriched in actives
200 motifs enriched in positives
200 motifs enriched in inactives
200 motifs enriched in negatives
Select top 10 motifs in each set
MEME-TOMTOM
108 motifs matched to 63 protein binding sites from databases (E-value<1)
56 motifs in both enhancers and non-enhancers
41 motifs enriched only in enhancers
11 motifs enriched only in non-enhancers
(B)
(C)
Figure S8: Identification of motifs enriched in the TAL1 bound enhancers and inactive segments. (A) The overall procedure for identifying TF binding site motifs that contributes to discrimination of TAL1 OSs by activity. (B) Distribution of G+C content (%) in four data sets: Enhancers and inactive regions identified in transient transfection assays, and positives and negatives from transgenic mice assays. (C) Frequency of 200 motifs defined by DME2. The top 10 motifs (5% of 200 motifs) were used to run further analyses (Tables S10-S13 in Additional file 2).

## Slide 9
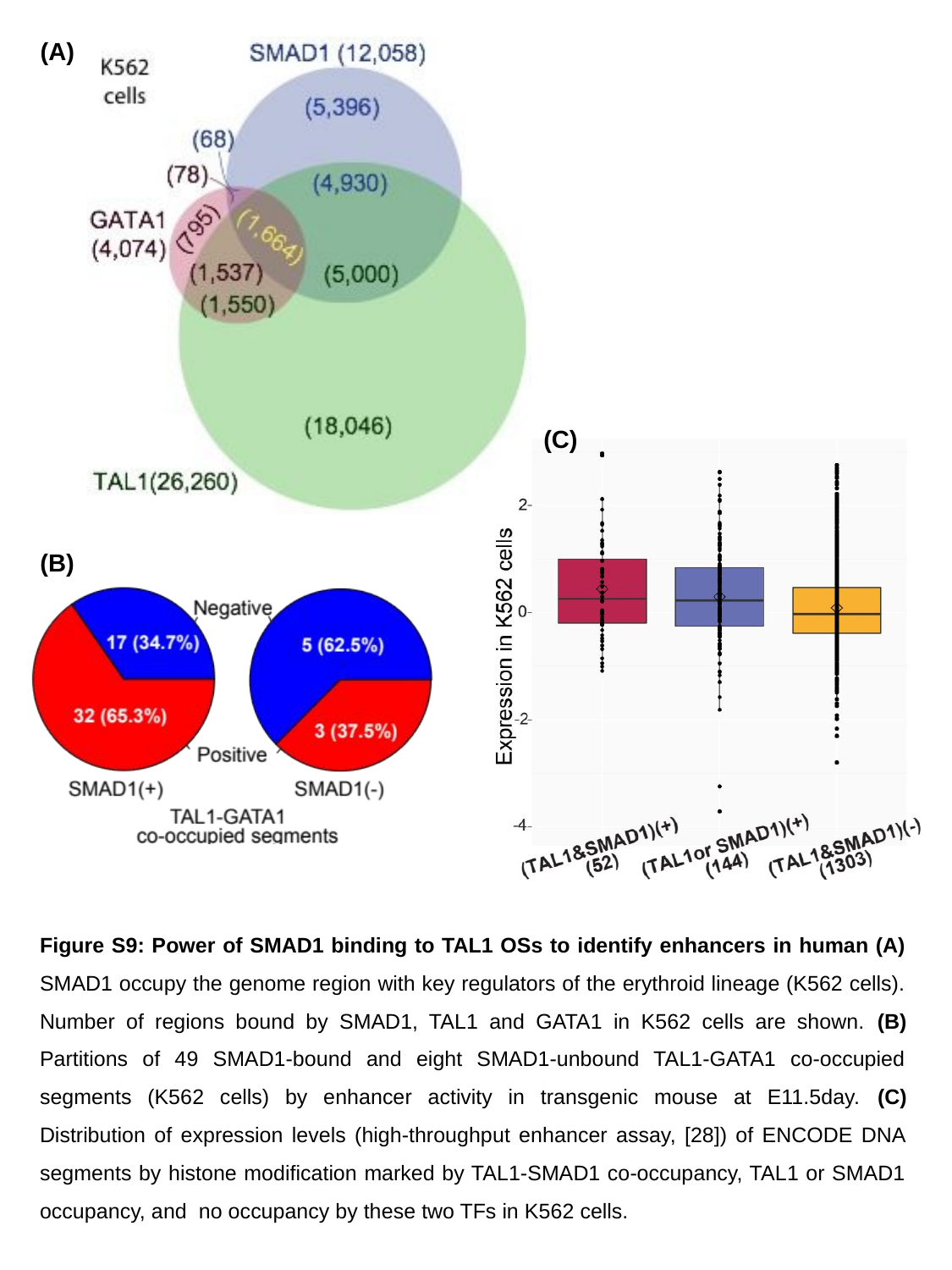

(A)
(C)
(B)
Figure S9: Power of SMAD1 binding to TAL1 OSs to identify enhancers in human (A) SMAD1 occupy the genome region with key regulators of the erythroid lineage (K562 cells). Number of regions bound by SMAD1, TAL1 and GATA1 in K562 cells are shown. (B) Partitions of 49 SMAD1-bound and eight SMAD1-unbound TAL1-GATA1 co-occupied segments (K562 cells) by enhancer activity in transgenic mouse at E11.5day. (C) Distribution of expression levels (high-throughput enhancer assay, [28]) of ENCODE DNA segments by histone modification marked by TAL1-SMAD1 co-occupancy, TAL1 or SMAD1 occupancy, and no occupancy by these two TFs in K562 cells.

## Slide 10
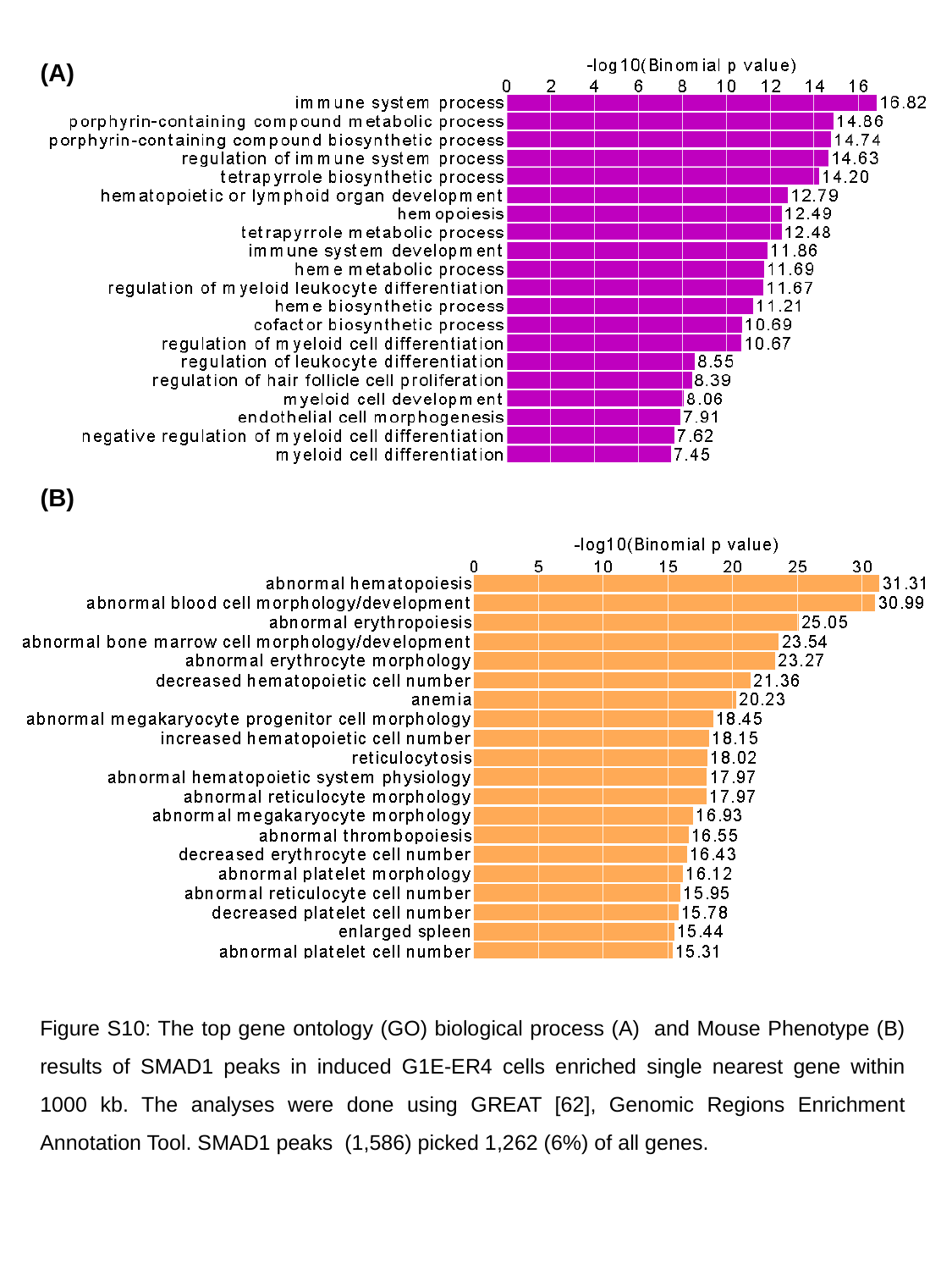

(A)
(B)
Figure S10: The top gene ontology (GO) biological process (A) and Mouse Phenotype (B) results of SMAD1 peaks in induced G1E-ER4 cells enriched single nearest gene within 1000 kb. The analyses were done using GREAT [62], Genomic Regions Enrichment Annotation Tool. SMAD1 peaks (1,586) picked 1,262 (6%) of all genes.

## Slide 11
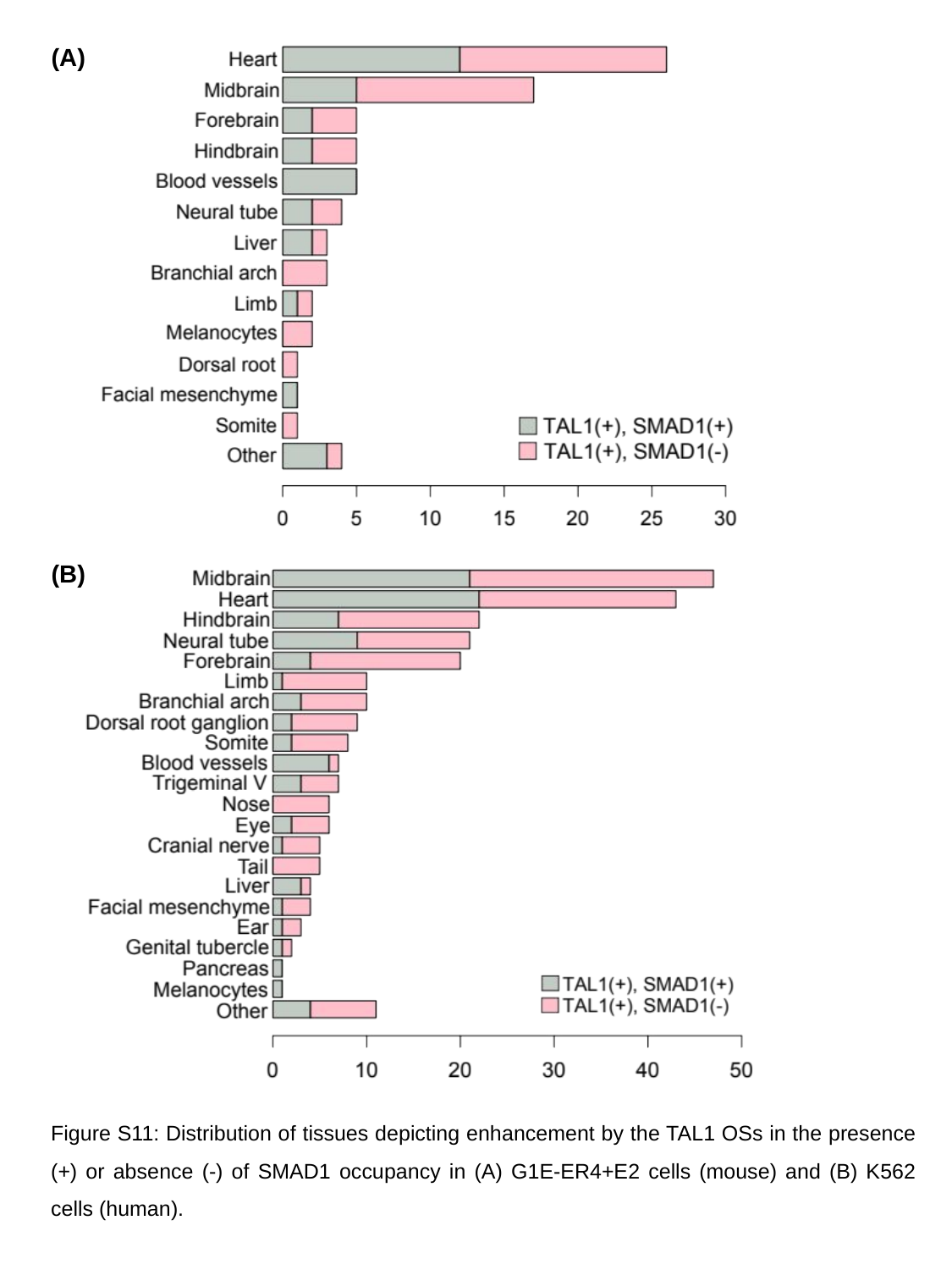

(A)
(B)
Figure S11: Distribution of tissues depicting enhancement by the TAL1 OSs in the presence (+) or absence (-) of SMAD1 occupancy in (A) G1E-ER4+E2 cells (mouse) and (B) K562 cells (human).

## Slide 12
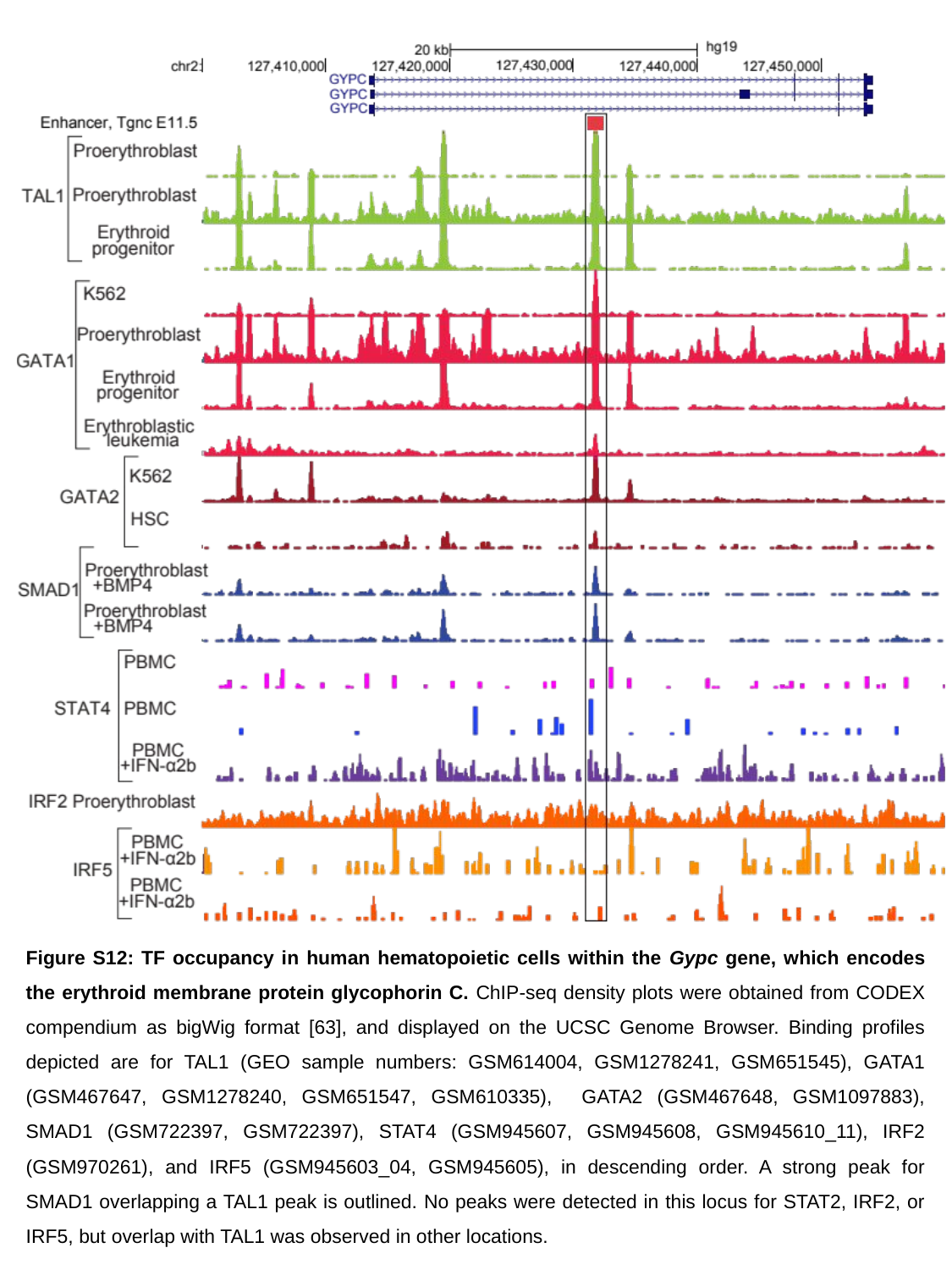

Figure S12: TF occupancy in human hematopoietic cells within the Gypc gene, which encodes the erythroid membrane protein glycophorin C. ChIP-seq density plots were obtained from CODEX compendium as bigWig format [63], and displayed on the UCSC Genome Browser. Binding profiles depicted are for TAL1 (GEO sample numbers: GSM614004, GSM1278241, GSM651545), GATA1 (GSM467647, GSM1278240, GSM651547, GSM610335), GATA2 (GSM467648, GSM1097883), SMAD1 (GSM722397, GSM722397), STAT4 (GSM945607, GSM945608, GSM945610_11), IRF2 (GSM970261), and IRF5 (GSM945603_04, GSM945605), in descending order. A strong peak for SMAD1 overlapping a TAL1 peak is outlined. No peaks were detected in this locus for STAT2, IRF2, or IRF5, but overlap with TAL1 was observed in other locations.

## Slide 13
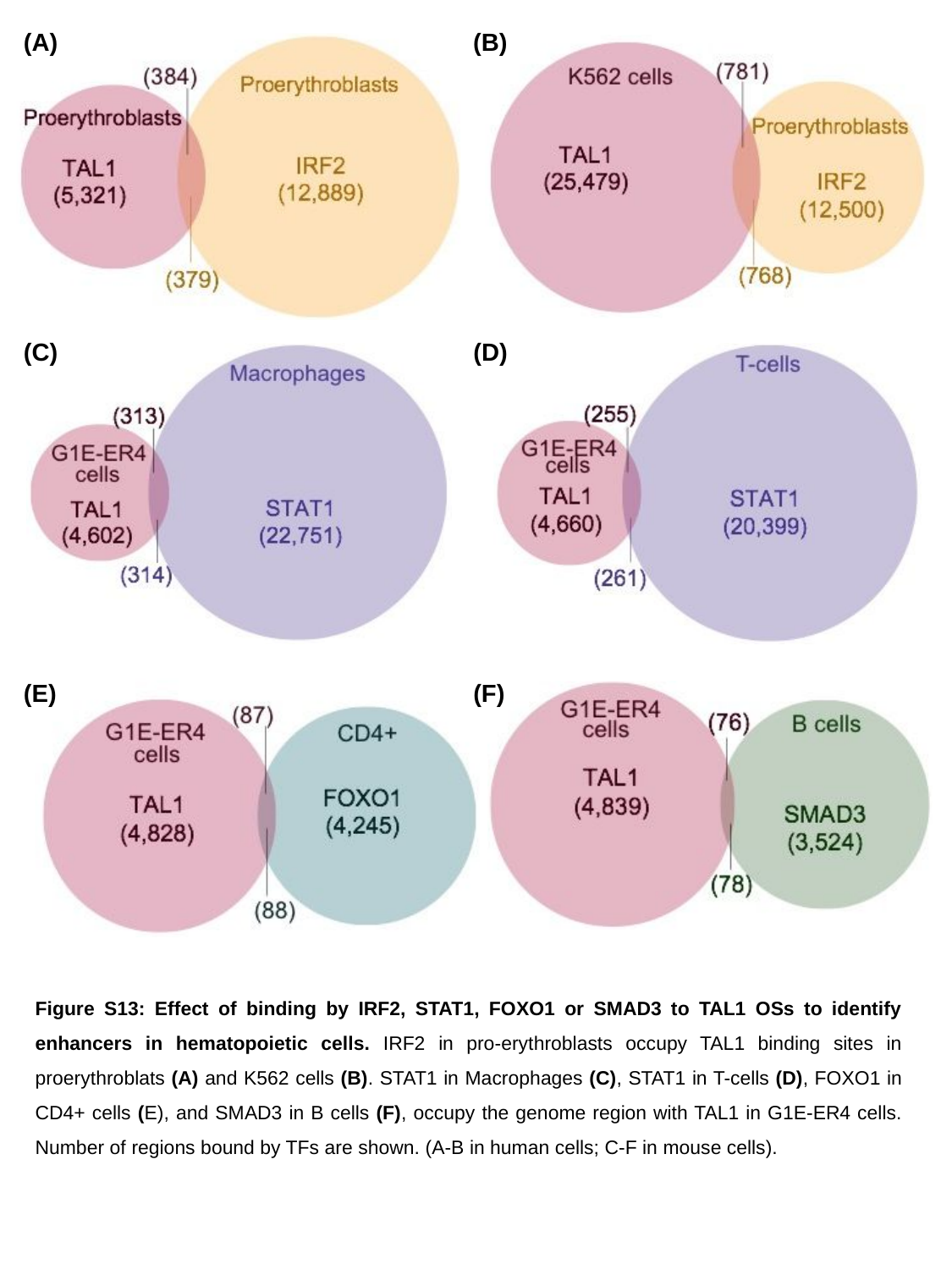

(A)
(B)
(C)
(D)
(E)
(F)
Figure S13: Effect of binding by IRF2, STAT1, FOXO1 or SMAD3 to TAL1 OSs to identify enhancers in hematopoietic cells. IRF2 in pro-erythroblasts occupy TAL1 binding sites in proerythroblats (A) and K562 cells (B). STAT1 in Macrophages (C), STAT1 in T-cells (D), FOXO1 in CD4+ cells (E), and SMAD3 in B cells (F), occupy the genome region with TAL1 in G1E-ER4 cells. Number of regions bound by TFs are shown. (A-B in human cells; C-F in mouse cells).

## Slide 14
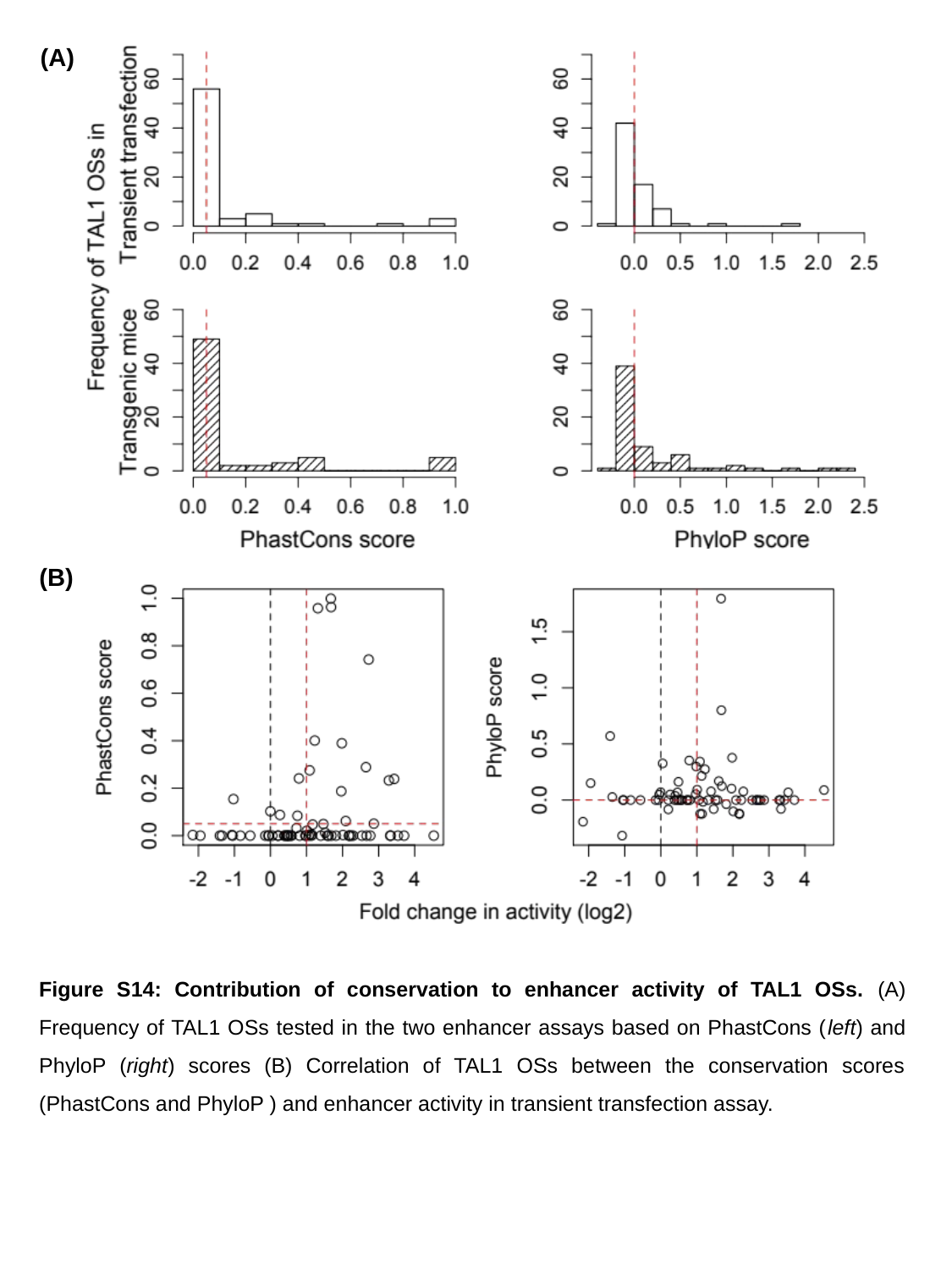

(A)
(B)
Figure S14: Contribution of conservation to enhancer activity of TAL1 OSs. (A) Frequency of TAL1 OSs tested in the two enhancer assays based on PhastCons (left) and PhyloP (right) scores (B) Correlation of TAL1 OSs between the conservation scores (PhastCons and PhyloP ) and enhancer activity in transient transfection assay.

## Slide 15
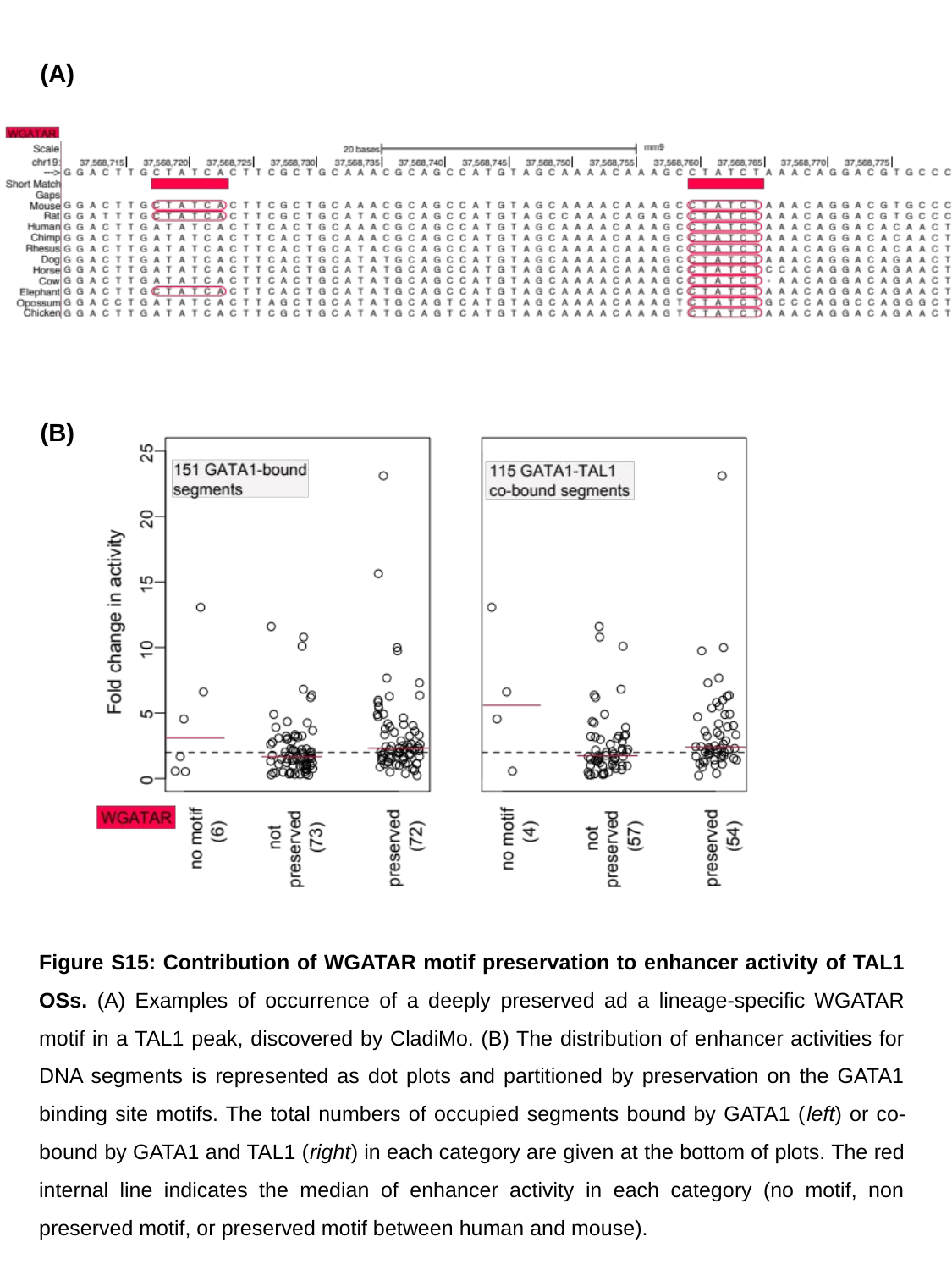

(A)
(B)
Figure S15: Contribution of WGATAR motif preservation to enhancer activity of TAL1 OSs. (A) Examples of occurrence of a deeply preserved ad a lineage-specific WGATAR motif in a TAL1 peak, discovered by CladiMo. (B) The distribution of enhancer activities for DNA segments is represented as dot plots and partitioned by preservation on the GATA1 binding site motifs. The total numbers of occupied segments bound by GATA1 (left) or co-bound by GATA1 and TAL1 (right) in each category are given at the bottom of plots. The red internal line indicates the median of enhancer activity in each category (no motif, non preserved motif, or preserved motif between human and mouse).
